# Semen miRNAs Contained in Exosomes as Non-Invasive Biomarkers for Prostate Cancer Diagnosis

**DOI:** 10.1038/s41598-019-50172-6

**Published:** 2019-09-24

**Authors:** Maria Barceló, Manel Castells, Lluís Bassas, Francesc Vigués, Sara Larriba

**Affiliations:** 1 Human Molecular Genetics Group- Bellvitge Biomedical Research Institute (IDIBELL), 08908 Hospitalet de Llobregat, Barcelona, Spain; 2 Urology Service, Bellvitge University Hospital‐ICS, 08908 Hospitalet de Llobregat, Barcelona, Spain; 3 Laboratory of Seminology and Embryology, Andrology Service-Fundació Puigvert, 08025 Barcelona, Spain

**Keywords:** Prostate cancer, miRNAs, Diagnostic markers, Prostate

## Abstract

Although it is specific for prostatic tissue, serum prostate-specific antigen (PSA) screening has resulted in an over-diagnosis of prostate cancer (PCa) and many unnecessary biopsies of benign disease due to a well-documented low cancer specificity, thus improvement is required. We profiled the expression level of miRNAs contained in semen exosomes from men with moderately increased PSA levels to assess their usefulness, either alone or in addition to PSA marker, as non-invasive biomarkers, for the early efficient diagnosis and prognosis of PCa. An altered miRNA expression pattern was found by a high throughput profiling analysis in PCa when compared with healthy individuals (HCt) exosomal semen samples. The presence of vasectomy was taken into account for the interpretation of results. Fourteen miRNAs were selected for miRNA validation as PCa biomarkers in a subsequent set of semen samples. In this explorative study, we describe miRNA-based models, which included miRNA expression values together with PSA levels, that increased the classification function of the PSA screening test with diagnostic and/or prognostic potential: [PSA + miR-142-3p + miR-142-5p + miR-223-3p] model (AUC:0,821) to discriminate PCa from BPH (Sn:91,7% Sp:42,9% vs Sn:100% Sp:14,3%); and [PSA + miR-342-3p + miR-374b-5p] model (AUC: 0,891) to discriminate between GS ≥ 7 tumours and men presenting PSA ≥ 4 ng/ml with no cancer or GS6 tumours (Sn:81,8% Sp:95% vs Sn:54,5% Sp:90%). The pathway analysis of predicted miRNA target genes supports a role for these miRNAs in PCa aetiology and/or progression. Our study shows semen exosome miRNA-based models as molecular biomarkers with the potential to improve PCa diagnosis/prognosis efficiency. As the next step, further prospective studies on larger cohorts of patients are required to validate the diagnostic and/or prognostic role of the miRNA panel before it could be adopted into clinical practice.

## Introduction

Prostate cancer (PCa) is the most prevalent type of malignant male cancer in Western countries and a major cause of cancer-related deaths. Detection is mainly carried out by the determination of levels of prostate-specific antigen (PSA) in blood and/or by physical examination of the prostate gland (digital rectal examination –DRE-). Suspicious results are evaluated in prostate tissue samples (transrectal or transperineal biopsy), essential to confirm the diagnosis and in which the severity or degree of affectation will be determined by means of the modified Gleason Score (GS)^1^.

PCa is a slow-growing tumour and fortunately it progresses to invasive PCa in only a minority of these patients. However, it may not cause signs or symptoms in its early stages, so it is critical to specifically identify those patients with clinically significant PCa and at an early stage (reviewed in^2^). Since its application in clinical practice, the screening of PCa based on the determination of PSA has allowed for better detection of the disease in the early stages, and therefore it has contributed to the reduction of mortality due to malignant prostate disease. However, the deficiencies of serum PSA as a biomarker are well documented^3^. Although specific for prostatic tissue, PSA has low cancer specificity^4^. Thus, PSA screening has resulted in an over-diagnosis of PCa, and in many unnecessary biopsies of benign disease; only about 30–40% of the biopsied men are diagnosed with PCa and specifically, in patients with PSA levels of 4 to 10 ng/ml, the detection rate of PCa was merely 20% or less thus defining the region as a “grey zone”. Conversely, not all prostate cancers give rise to an elevated serum PSA concentration^4^. Additionally, serum PSA levels do not correlate with tumour aggressiveness, survival, or response to pharmacological treatments leading to over-treatment of indolent tumours. Given this context, more specific non-invasive diagnostic biomarkers, either alone or in addition to PSA marker, that could identify PCa patients would be very welcomed indeed.

Approximately 40% of semen is derived from prostatic tissue, so that its contents are most likely to contain prostate disease-specific derived molecules which can be potentially used as PCa-specific biomarkers^5–7^. Interestingly, seminal plasma (SP) contains an extraordinary concentration of extracellular miRNAs, some of them specific to the reproductive glands where it originates, such as the prostate. MicroRNAs (miRNAs) are conserved small non-coding RNAs (19–22 nucleotide-long) that mediate post-transcriptional regulation of gene expression, affecting mRNA stability and translation by binding complementary sites on target mRNAs^8^. Studies have shown that the miRNAs have critical roles in a variety of biological processes such as: cell proliferation, differentiation, apoptosis and carcinogenesis^9,10^. Interestingly, some molecular studies have shown a fingerprint of altered miRNA expression in FFPE prostate tumour tissue compared with benign disease controls^11^, suggesting a diagnostic potential of miRNAs as biomarkers for PCa.

Such extracellular miRNAs in biofluids, and specifically in semen, are very stable and resistant to endogenous ribonuclease activity due to their presentation, either contained in cell-derived extracellular vesicles (EVs) such as exosomes^12^ or free/soluble in protein complexes bound with RNA-binding proteins^13^. In addition to a high content of cholesterol and sphingomyelin, and a very complex protein composition, the exosomes contain coding and non-coding RNAs^12^ that can be transferred to recipient cells to modulate their function, thus mediating paracrine signalling^12,14^. Exosomes are released to various biological fluids and specifically, it has been calculated that, in mammals, each ejaculate contains trillions of exosomes. Exosomes have a role in immune regulation^15^, which is relevant in the genital mucosa.

The different organs of the male reproductive system secrete exosomes that will be part of the SP^16^. Prostasomes, originated from the prostate, are the main and the best studied exosomes in SP, they are secreted by epithelial, normal and pathological cells, and travel from the prostate gland to the seminal fluid. Prostasomes, and in general the exosomes in semen, have multiple physiological functions most of them related to the fertility process^17–20^. Additionally, both neoplastic and metastatic PCa cells have been shown to release prostasomes^21–23^, contributing to the spread and development of PCa.

In the present study we explored the potential of exosome miRNAs in semen from men with moderately altered serum PSA levels as non-invasive biomarkers of PCa.

Whether the subject has undergone vasectomy is taken into account for the accurate interpretation of results. The identification of non-invasive biomarkers for PCa would additionally help to determine the pathophysiological aetiology of PCa and it would enable the prediction of the presence of malignant PCa cells, which would provide an earlier diagnosis of the disease.

## Results

### Benign hyperplasia and malignant prostate tumour show altered exosomal miRNA profile in semen

The clinicopathological characteristics of patients are presented in Table 1 and Supplementary Table S1. Cases (PCa) and benign prostatic hyperplasia (BPH) controls mostly (25 out of 31 individuals: 80,6%) fall within the serum PSA diagnostic “grey zone” (4–10 ng/ml). These two groups were similar regarding age, whereas pre-biopsy PSA in PCa-noV was significantly increased (*p* = 0,02) when compared to BPH controls. The mean age of the healthy controls (HCt) differed significantly from the other groups (*p* < 0,001). Most cases (23 out of the 24 individuals) showed mild disease, with PCa in early stages (GS 6 or 7).

**Table 1:** Clinical details of individuals included in this study.

Variable	miRNA screening study				miRNA testing/validation study			
	HCt	BPH	PCa-noV	PCa-V	HCt	BPH	PCa-noV	PCa-V
Total, n	3	3	3	3	8	7	16	8
Age, mean ± SD (years)	40 ± 4,58	64 ± 3,00	60 ± 1,73	60,33 ± 9,07	41 ± 3,12	59,86 ± 4,70	58,87 ± 4,94	58,62 ± 9,08
Pre-biopsy PSA (n)								
≤10 (ng/ml)	3	3	3	3	*8*	7	13	5
>10 (ng/ml)	0	0	0	0	*0*	0	3	3
Pre-biopsy PSA, mean ± SD (ng/ml)	nd	4,75 ± 0,20	4,77 ± 0,46	5,00 ± 0,81	nd	4,66 ± 1,45	7,59 ± 3,60	8,35 ± 4,78
**Gleason score biopsy (n)**								
6 (3 + 3)	nd	nd	2	3	nd	nd	8	5
7 (3 + 4)	nd	nd	1	0	nd	nd	4	3
7 (4 + 3)	nd	nd	0	0	nd	nd	3	0
8 (4 + 4)	nd	nd	0	0	nd	nd	1	0
**Clinical stage (n)**								
T1c	nd	nd	2	2	nd	nd	11	3
T2a	nd	nd	0	0	nd	nd	0	1
T2c	nd	nd	1	1	nd	nd	3	3
T3a	nd	nd	0	0	nd	nd	2	1

HCt: healthy control group; BPH: benign prostate hyperplasia group; PCa-noV: prostate cancer from non-vasectomized individuals; PCa-V: prostate cancer from vasectomised individuals.

Text in italics refers to healthy individuals that were not analysed for PSA. In this case, PSA levels were inferred from PSA reference values of healthy men based on age (Oesterling JE *et al*., 1993)^44^.

**Figure 1:**
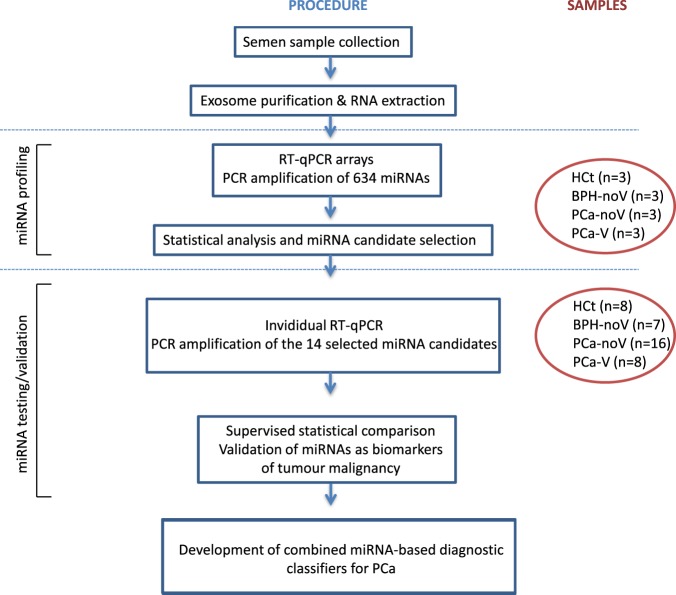
Flow chart representing the two stages design study. The number of miRNAs and individuals analysed in each work-procedure stage are depicted.

The research is conceived as an assessment/development pilot study for a PCa biomarker test based on semen exosome miRNAs which was divided into two phases (Fig. 1). In the first stage of the study, a high throughput analysis of the level of expression of 634 human miRNAs using a low number of study subjects was performed in order to identify the global exosomal miRNA changes in seminal fluid associated with malignant PCa. All the BPH and PCa individuals included in this first phase presented PSA levels within the 4–6 ng/ml range (Supplementary Table S1).

We were able to statistically analyze the expression behaviour of 400 miRNAs (63%) between groups (Supplementary Table S5).The rest of the miRNAs were excluded from analysis as in some cases (n = 165) no amplification values (Cp value > 38) were obtained in either group of the study, suggesting that the transcript levels of these miRNAs were beneath the detection threshold of the technique, and in other cases (n = 69) poor amplification efficiency across samples (missing expression values for eight out of the twelve samples) was obtained (data not shown).

The presence of 392 miRNAs was confirmed in HCt samples; 42 of these miRNAs were not expressed in PCa-V samples, although they presented detectable expression in PCa-noV, suggesting that they are preferentially expressed in testis and/or epididymis.

Our study revealed significantly altered expression levels of 100 miRNAs in PCa and/or BPH compared with HCt individuals (uncorrected *p* ≤ 0,05) (Table 2). Fifty of them were significantly under-expressed in PCa-V individuals, but not in PCa-noV (Table 2; miRNAs in the last positions in the table), suggesting that the loss of expression is a consequence of the vasectomy procedure and does not originate from the presence of the tumour. As previously described in obstructive azoospermia^24^, many of these miRNAs that present a reduced in expression in semen from vasectomised PCa individuals map to miRNA clusters in chromosome 19 and chromosome X (Table 2).

**Table 2:** Semen exosome-derived miRNAs differentially expressed in PCa and/or BPH compared with HCt individuals in the miRNA screening phase of the study

miRNA	Location	Seminal plasma exosomal miRNA expression			
		HCt	BPH-noV	PCa-noV	PCa-V
**A**. **Overexpressed miRNAs**					
***hsa-miR-142-5p***	17	1	**26**,675**	**17**,679**	**54**,632*
hsa-miR-520h^2*c*19,3*c*19^	19	1	**40**,285**	**20**,862*	**1**,**713**
hsa-miR-873-5p	9	1	**15**,860**	**18**,166*	**1**,**713**
hsa-miR-520g-3p^1*c*19,4*c*19^	19	1	**16**,149*	**20**,866*	**1**,**713**
*hsa-miR-513b-5p* ^3cX^	X	1	**6**,658*	**3**,854*	0,974
*hsa-miR-455-5p*	9	1	**3**,523*	**2**,902*	0,910
***hsa-miR-128-3p***	2, 3	1	**1**,595*	**1**,775*	1,273
***hsa-miR-142-3p***	17	1	**13**,047*	**5**,**214**	**13**,273*
***hsa-miR-223-3p***	X	1	**20**,124*	**8**,**926**	**14**,319*
***hsa-miR-212-5p***	17	1	**1**,**515**	**12**,505**	**14**,684*
***hsa-miR-182-3p***	7	1	**2**,**431**	**4**,102*	**2**,931*
***hsa-miR-130a-3p***	11	1	**2**,256**	**1**,**520**	1,324
hsa-miR-890^1cX^	X	1	**3**,850*	**1**,**655**	**0**,**010**
*hsa-miR-216a-5p*	2	1	**3**,716*	1,139	**1**,**631**
***hsa-miR-222-3p*** ^5cX^	X	1	**3**,697*	1,318	**0**,**267**
*hsa-miR-548k*	11	1	**3**,556*	**1**,**722**	0,878
*hsa-miR-31-5p*	9	1	**3**,141*	1,313	**0**,**373**
*hsa-miR-205-5p*	1	1	**3**,003*	1,106	**0**,**401**
hsa-miR-135b-5p	1	1	**2**,872*	1,177	**0**,**152**
*hsa-miR-181b-5p*	1, 9	1	**2**,844*	1,230	**0**,**295**
***hsa-miR-187-5p***	18	1	**2**,786*	**0**,**597**	**1**,**693**
*hsa-miR-187-3p*	18	1	**2**,646*	**1**,**595**	0,739
*hsa-miR-455-3p*	9	1	**2**,467*	**1**,**858**	**0**,**658**
*hsa-miR-500a-5p* ^6cX^	X	1	**2**,258*	0,752	**0**,**367**
*hsa-miR-877-5p*	6	1	**2**,113*	1,439	**0**,**376**
*hsa-miR-10b-5p*	2	1	**2**,007*	1,187	**0**,**446**
*hsa-miR-345-5p*	14	1	**1**,567*	1,100	0,901
*hsa-miR-96-5p*	7	1	**1**,531*	**1**,**766**	**1**,**403**
hsa-miR-15a-5p	13	1	1,364*	1,220	0,882
hsa-let-7i-5p	12	1	1,323*	1,041	0,891
***hsa-miR-370-3p***	14	1	**46**,**534**	**50**,733**	**30**,**243**
hsa-miR-9-3p	1, 5, 15	1	**13**,**047**	**16**,954*	**1**,**713**
*hsa-miR-376c-3p*	14	1	**3**,**286**	**3**,291*	**1**,**639**
hsa-miR-202-3p	10	1	0,970	**2**,271*	**0**,**010**
*hsa-miR-550a-3p*	7	1	**3**,**147**	**2**,150*	**2**,**371**
*hsa-miR-432-5p*	14	1	**1**,**560**	**1**,835*	**2**,**079**
*hsa-miR-145-5p*	5	1	**26**,**555**	**30**,**003**	**14**,812*
*hsa-miR-130b-5p*	22	1	**1**,**512**	1,022	**1**,939*
**B**. **Underexpressed miRNAs**					
***hsa-miR-342-3p***	14	1	**0**,606**	0,696*	**0**,610*
hsa-miR-151a-3p	8	1	0,842*	0,771*	0,720*
***hsa-miR-374b-5p***	X	1	**0**,599**	0,714	0,799*
*hsa-miR-125a-3p*	19	1	**0**,493*	0,818	**0**,460*
*hsa-miR-20a-5p*	13	1	0,727*	0,914	0,807*
***hsa-miR-217***	2	1	0,819	**0**,0582*	**0**,086*
*hsa-miR-582-3p*	5	1	**0**,009**	1,000	1,249
*hsa-miR-149-5p*	2	1	**0**,564**	0,806	0,677
hsa-miR-125b-2-3p	21	1	0,701**	0,927	**0**,**549**
hsa-miR-365a-3p	16	1	0,793**	1,135	1,126
***hsa-miR-150-5p***	19	1	**0**,029*	1,292	1,013
*hsa-miR-99a-5p*	21	1	**0**,609*	0,978	**0**,**649**
hsa-miR-30b-5p	8	1	0,748*	0,884	0,926
hsa-miR-191-5p	3	1	0,770*	0,907	0,861
hsa-miR-18b-5p	X	1	0,882*	0,940	0,889
*hsa-miR-369-3p*	14	1	1,281	**0**,066**	0,954
***hsa-miR-425-3p***	3	1	0,969	0,695*	0,786
hsa-miR-193a-5p	17	1	0,799	0,742*	0,773
hsa-miR-892a^1cX^	X	1	**2**,**127**	0,877	**0**,002**
hsa-miR-514a-3p^2cX^	X	1	**2**,**052**	1,234	**0**,003**
hsa-miR-34b-5p	11	1	**3**,**515**	**2**,**953**	**0**,005**
hsa-miR-888-5p^1cX^	X	1	**2**,**682**	1,207	**0**,009**
hsa-miR-202-3p	10	1	0,970	**2**,**271**	**0**,010**
hsa-miR-509-3-5p	X	1	1,296	1,282	**0**,011**
hsa-miR-513c-5p^3cX^	X	1	**2**,**202**	**1**,**811**	**0**,015**
hsa-miR-34b-3p	11	1	1,240	1,173	**0**,017**
hsa-miR-517a-3p^1*c*19^	19	1	1,081	1,384	**0**,022**
hsa-miR-34c-5p	11	1	**3**,**325**	**1**,**772**	**0**,028**
hsa-miR-517c-3p^2*c*19^	19	1	1,134	1,379	**0**,037**
hsa-miR-551b-3p	3	1	**1**,**506**	1,111	**0**,039**
hsa-miR-888-3p^1cX^	X	1	1,465	0,967	**0**,054**
hsa-miR-507^4cX^	X	1	1,197	1,125	**0**,055**
hsa-miR-519a-3p^3*c*19^	19	1	1,199	**2**,**194**	**0**,083**
hsa-miR-519d-3p^1*c*19^	19	1	**1**,**755**	1,451	**0**,085**
hsa-miR-135b-5p	1	1	**2**,**872**	1,177	**0**,152**
hsa-miR-221-3p^5cX^	X	1	**2**,**443**	1,007	**0**,197**
hsa-miR-532-5p^6cX^	X	1	1,067	0,982	0,679**
hsa-miR-891a-5p	X	1	**2**,**737**	1,197	**0**,012*
hsa-miR-891b^1cX^	X	1	**2**,**861**	1,397	**0**,024*
hsa-miR-424-3p^7cX^	X	1	**3**,**023**	1,110	**0**,035*
hsa-miR-508-3p^4cX^	X	1	**2**,**098**	1,264	**0**,051*
hsa-miR-450a-5p^7cX^	X	1	**2**,**862**	**1**,**567**	**0**,054*
hsa-miR-1537-3p	1	1	0,791	**0**,**557**	**0**,058*
hsa-miR-510-5p^2cX^	X	1	**5**,**228**	**3**,**614**	**0**,064*
hsa-miR-518e-5p^4*c*19^	19	1	1,443	1,232	**0**,080*
hsa-miR-449a	5	1	**1**,**839**	**2**,**002**	**0**,116*
hsa-miR-522-3p^2*c*19,3*c*19^	19	1	**2**,**539**	**3**,**910**	**0**,127*
hsa-miR-376a-5p	14	1	**1**,**576**	1,340	**0**,133*
hsa-miR-513a-3p^4cX^	X	1	**2**,**228**	1,378	**0**,139*
hsa-miR-518e-3p^4*c*19^	19	1	**2**,**023**	**3**,**256**	**0**,146*
hsa-miR-506-3p^4cX^	X	1	**2**,**423**	1,206	**0**,153*
hsa-miR-382-3p	14	1	**2**,**191**	1,386	**0**,168*
hsa-miR-10a-5p	17	1	**2**,**207**	0,742	**0**,212*
hsa-miR-508-5p^4cX^	X	1	**7**,**228**	**2**,**926**	**0**,254*
hsa-miR-222-3p^5cX^	X	1	**3**,**697**	1,318	**0**,267*
hsa-miR-181b-5p	1, 9	1	**2**,**844**	1,230	**0**,295*
hsa-miR-146b-5p	10	1	**1**,**765**	1,177	**0**,331*
hsa-miR-500a-5p^6cX^	X	1	**2**,**258**	0,752	**0**,367*
hsa-miR-31-5p	9	1	**3**,**141**	1,313	**0**,373*
hsa-miR-181a-5p	1, 9	1	**1**,**887**	0,996	**0**,384*
hsa-miR-204-5p	9	1	**1**,**736**	1,163	**0**,471*
hsa-miR-452-5p	X	1	0,940	1,01	**0**,500*
hsa-miR-502-3p^6cX^	X	1	**1**,**874**	1,213	**0**,584*
hsa-miR-660-5p^6cX^	X	1	1,463	1,012	**0**,585*
hsa-miR-532-3p^6cX^	X	1	0,834	0,892	**0**,618*
hsa-miR-200b-5p	1	1	0,767	0,809	0,679*
hsa-miR-200b-3p	1	1	0,877	0,893	0,710*
hsa-miR-103a-3p	5, 20	1	0,890	0,969	0,845*

Statistically altered miRNA expression levels are pointed with asterisks, when compared with HCt controls;*p ≤ 0,05; **p ≤ 0,005.

1,5-fold increase or decrease compared to HCt control is depicted in bold.

1-7cX clusters in chromosome X.

1-4c19 clusters in chromosome 19.

miRNAs that fulfilled the criteria for testing/validation (≥1,5 fold difference in expression between groups, a Cp value ≤ 36 in any of the groups, and additionally, the expression in PCa-V should represent >20% of PCa-noV expression) are depicted in italics. miRNAs selected for miRNA testing/validation are depicted in italics and bold.

Among the remaining differentially expressed miRNAs (Table 2), 42 of them were altered in expression in BPH when compared to HCt controls (28 over-expressed and 14 under-expressed); 21 miRNAs were dysregulated in PCa-noV compared to HCt controls (15 over-expressed and 6 under-expressed); six miRNAs were shared among PCa-noV and PCa-V (either up-regulated miR-142-5p, miR-212-5p, miR-182-3p, or down-regulated miR-342-3p, miR-151a-3p, miR-217), and three of them were shared among BPH and PCa samples (miR-142-5p, miR-151a-3p, miR-342-3p). Additionally, when PCa were compared to BPH semen samples we found 11 up-regulated and 16 down-regulated miRNAs (Table 3). Nevertheless, no miRNA passed the 5% FDR correction (*p* ≤ 0,0001).

**Table 3:** Semen exosome-derived miRNAs differentially expressed in PCa compared with BPH individuals in the miRNA screening phase of the study.

miRNA	Location	Seminal plasma exosomal miRNA expression		
		BPH-noV	PCa-noV	PCa-V
**A**. **Overexpressed miRNAs**				
hsa-miR-503-5p		1	**11**,856*	1,131
hsa-miR-199b-5p		1	**11**,181**	1,131
hsa-miR-212-5p		1	**8**,255**	**9**,694**
hsa-miR-200c-5p		1	**1**,840**	1,140
hsa-miR-99a-5p		1	**1**,605*	1,065
hsa-miR-99a-3p		1	**1**,587*	1,083
hsa-miR-574-3p		1	1,383*	1,479**
hsa-miR-664a-3p		1	1,364*	1,133
hsa-miR-196b-5p		1	1,299*	1,235
hsa-miR-20a-5p		1	1,257*	1,110
hsa-miR-454-3p		1	1,224*	0,996
**B**. **Underexpressed miRNAs**				
hsa-miR-139-5p		1	**0**,031*	0,778
hsa-miR-205-3p		1	**0**,034**	**0**,050**
hsa-miR-217		1	**0**,071**	**0**,105**
hsa-miR-485-3p		1	**0**,184*	**0**,**268**
hsa-miR-187-5p		1	**0**,214*	**0**,**608**
hsa-miR-500a-5p		1	**0**,333*	**0**,163**
hsa-miR-222-3p		1	**0**,356*	**0**,072**
hsa-miR-205-5p		1	**0**,368*	**0**,134**
hsa-miR-92b-3p		1	**0**,404**	**0**,**238**
hsa-miR-135b-5p		1	**0**,410*	**0**,053**
hsa-miR-10a-3p		1	**0**,410*	**0**,043**
hsa-miR-31-5p		1	**0**,418*	**0**,119**
hsa-miR-181b-5p		1	**0**,432*	**0**,104**
hsa-miR-31-3p		1	**0**,494**	**0**,**137**
hsa-miR-493-5p		1	**0**,633*	**0**,050**
hsa-miR-425-3p		1	0,717*	0,812

Statistically altered miRNA expression levels are pointed with asterisks, when compared with BPH controls;

*p < 0,05; **p < 0,005.

1,5-fold increase or decrease compared to HCt control is depicted in bold.

Given the profiling results in the miRNA panels, we proceeded to test/validate several miRNAs as candidate biomarkers of PCa malignancy in a larger cohort of samples (second phase of the study; Fig. 1). Several miRNAs were candidates for validation based on the following criteria: we selected those miRNAs that presented ≥1,5 fold difference in expression between groups as biologically and clinically relevant, a Cp value ≤ 36 in any of the groups, and additionally, the expression in PCa-V should represent >20% of expression in PCa-noV (Table 2, Supplementary Table S2). Out of 39 miRNAs that fulfilled the criteria, 14 were finally selected for miRNA validation (up-regulated: miR-142-5p, miR-128-3p, miR-142-3p, miR-223-3p, miR-212-5p, miR-182-3p, miR-130a-3p, miR-222-3p, miR-187-5p, miR-370-3p; down-regulated: miR-342-3p, miR-374b-5p, miR-217, miR-150-5p). Interestingly, these included five out of the six miRNAs deregulated in both PCa-noV and PCa-V samples and three miRNAs (miR-212-5p, miR-217, miR-222-3p) that presented significant differences in expression between PCa and BPH phenotypes whereas one miRNA (miR-187-5p) was differentially expressed between PCa-noV and BPH (Table 3).

**Figure 2:**
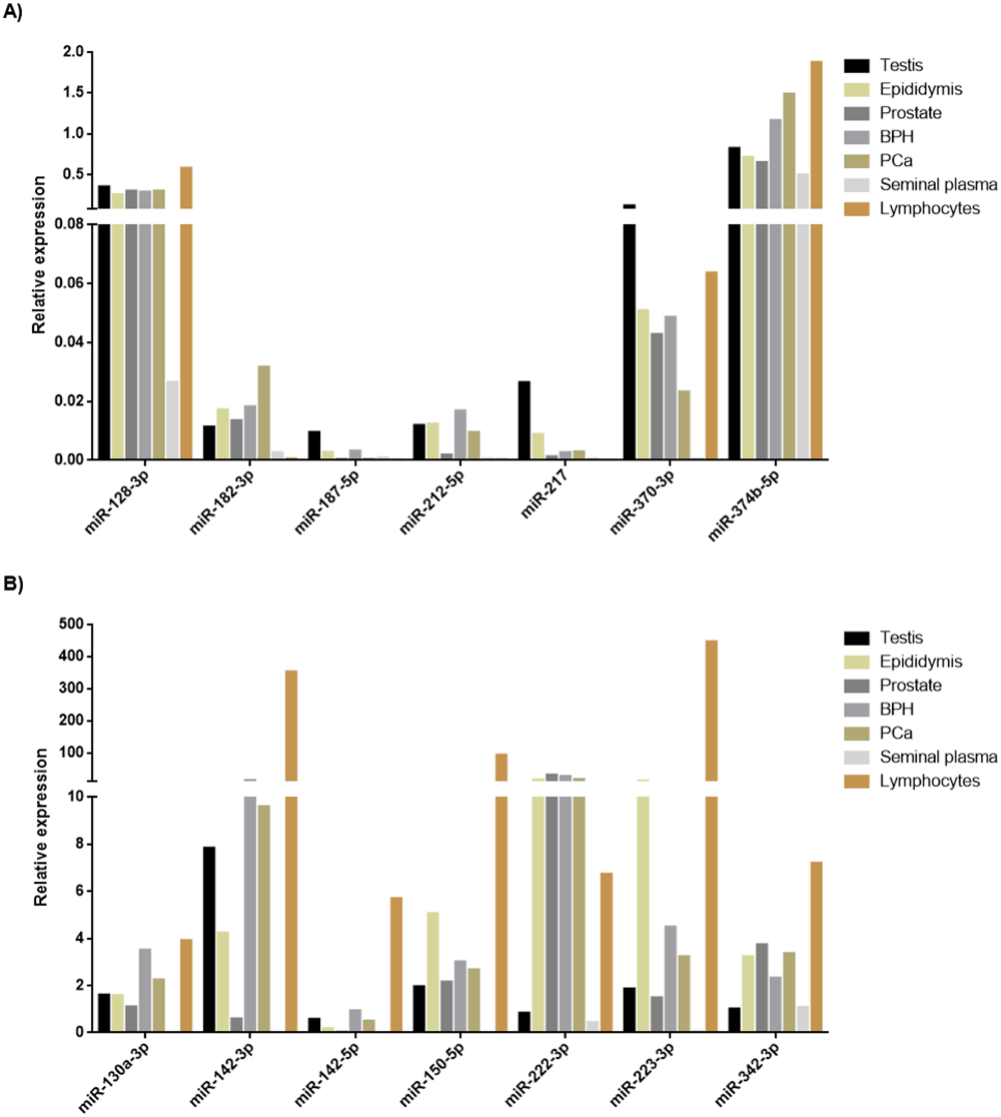
Tissue expression profiling of the 14 candidate miRNAs. miRNA expression was determined by RT-qPCR in several reproductive organs such as testis, epididymis and prostate, as well as in exosomes from seminal plasma (SP) and in lymphocytes. Controls of pathological prostate (benign prostate hyperplasia –BPH- and prostate cancer –PCa- prostate) were also included. Expression levels relative to miR-30e-3p and miR-126-3p are shown.

In order to determine the expression level of each miRNA in the different organs of the reproductive tract, the expression of the 14 miRNAs was first tested in testis, epididymis, prostate and SP (two samples each). Controls of pathological prostate (BPH and PCa tissue) and lymphocytes, the latter as external control cells, were also included (two samples each). We were able to corroborate that all miRNAs tested were expressed in the prostate and most of them were altered in expression in the BPH and PCa prostate samples (Fig. 2).

**Figure 3:**
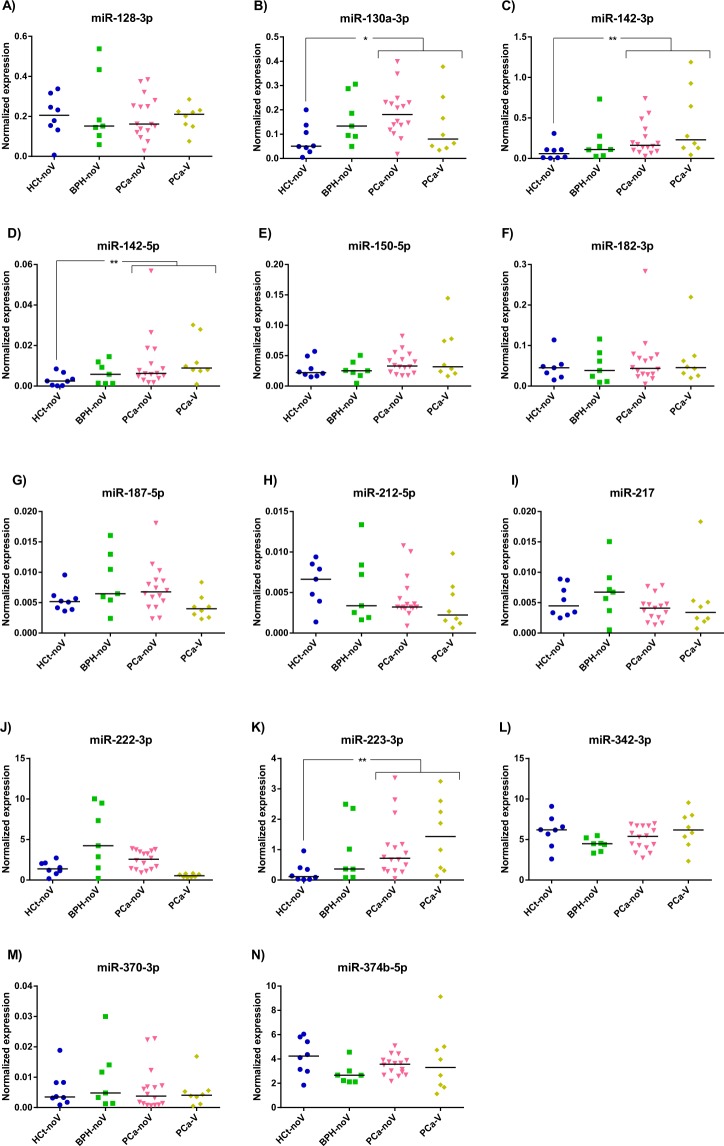
Exosome miRNA levels in SP are altered in benign prostate hyperplasia and malignant prostate tumour. Expression profiling, at the testing/validating stage, of the miRNAs in exosomes from semen of healthy controls (HCt), benign prostate hyperplasia-non vasectomised (BPH-noV), prostate cancer-non vasectomised (PCa-noV) and prostate cancer from men successfully vasectomised (PCa-V). The horizontal bar displays the median cellular expression level. Significant differences between groups are indicated: *p < 0,05; **p < 0,01 (Mann Whitney U test)

Therefore, in the miRNA testing/validation phase of the study (Fig. 1; Supplementary Table S1), these particular 14 miRNAs were individually reanalysed in a larger set of semen samples by RT-qPCR (Fig. 3). The expression tendencies between groups of the 14 miRNAs analysed were mostly conserved between the miRNA panels and the RT-qPCR individual assays (Supplementary Fig. S1).

### Diagnostic performance of single miRNAs and development of combined miRNA-based diagnostic classifiers for PCa

First, our validation results showed that the expression values of miR-130a-3p (*p* = 0,015), miR-142-3p (*p* = 0,006), miR-142-5p (*p* = 0,006) and miR-223-3p (*p* = 0,003) were statistically different between PCa and HCt groups (Fig. 3). Specifically, when PCa samples were divided into PCa-V and PCa-noV groups, miR-130a-3p (*p* = 0,005), miR-142-3p (*p* = 0,019), miR-142-5p (*p* = 0,019), miR-223-3p (*p* = 0,009) and miR-222-3p (*p* = 0,045) expression values were statistically different between PCa-noV and HCt groups, whereas miR-142-3p (*p* = 0,010), miR-142-5p (*p* = 0,010), miR-223-3p (*p* = 0,010) and miR-222-3p (*p* = 0,015) expression values were statistically different between PCa-V and HCt groups. Interestingly, three of these miRNAs were statistically different in expression in the presence of malignant tumour in the prostate (PCa group) when compared with the absence of a tumour (HCt + BPH group): miR-142-3p (*p* = 0,012), miR-142-5p (*p* = 0,015) and miR-223-3p (*p* = 0,020). Strikingly, we found that miR-142-3p, miR-142-5p and miR-223-3p were indeed over-expressed in PCa and BPH tissue when compared with healthy tissue (Fig. 2) suggesting that the over-expression of these miRNAs in prostate perfectly fits with a quantified higher concentration in semen exosomes and, thus may well be a reflection of the prostate health.

**Figure 4:**
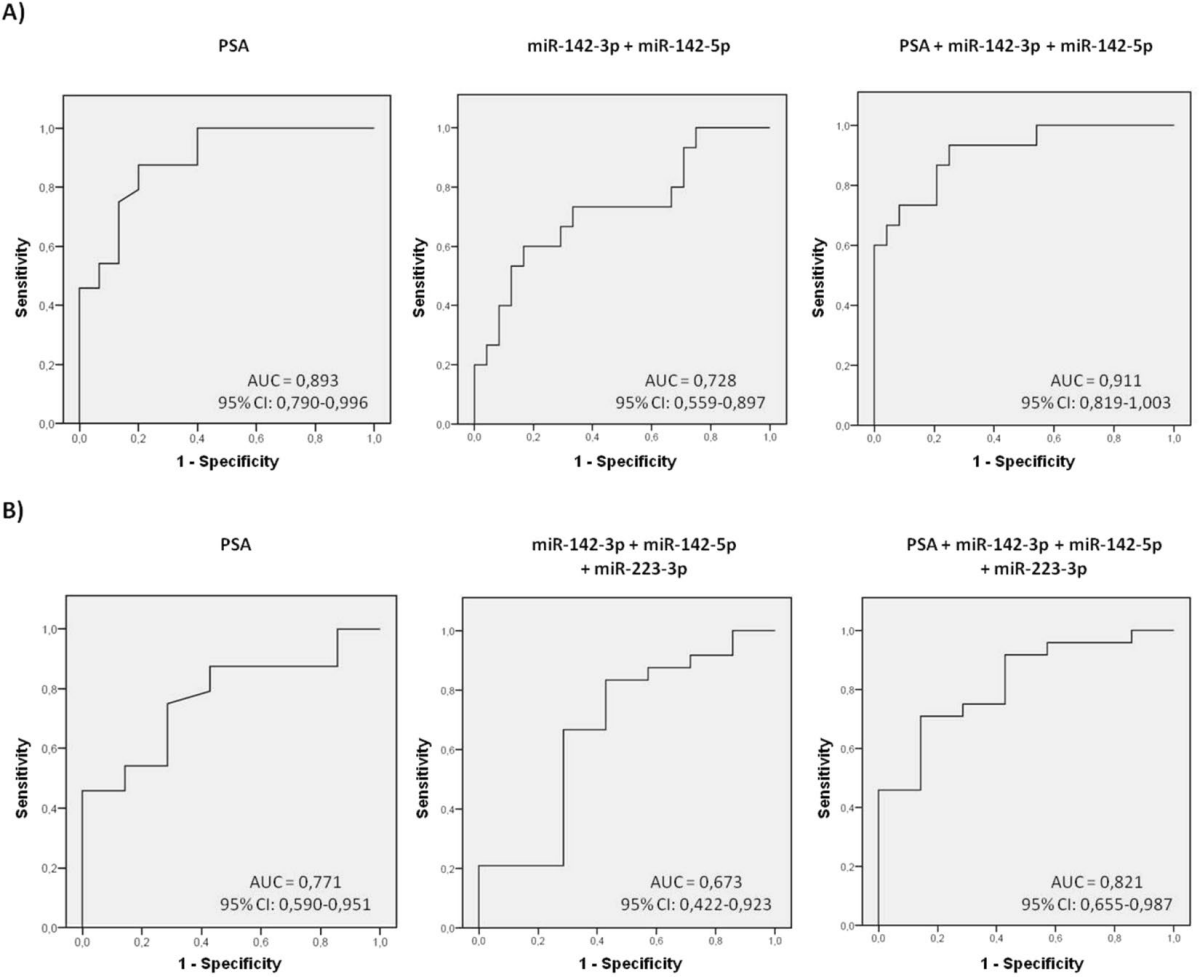
MiRNA-based models as diagnostic classifiers. Receiver operating characteristic (ROC) curves showing the predictive efficiency for distinguishing A) PCa from (HCt + BPH) and B) PCa from BPH samples, by using serum PSA, the model obtained from the combination of miRNAs (miR-142-3p, miR-142-5p and/or miR-223-3p) or the model that additionally includes PSA with the miRNAs (PSA, miR-142-3p, miR-142-5p and/or miR-223-3p) at the testing/validation stage. The multivariate models were obtained by performing a multivariate binary logistic regression analysis (backward stepwise, conditional method). AUC: area under the curve; 95% CI: a 95% of confidence interval.

The expression values of these three miRNAs in semen exosomes resulted in good predictive accuracy [miR-142-3p (AUC: 0,739, *p* = 0,013), miR-142-5p (AUC: 0,733, *p* = 0,015) and miR-223-3p (AUC: 0,722, *p* = 0,021)] to discriminate PCa from (HCt + BPH) control individuals, suggesting that they have a potential use as indicators of the presence of malignant cells in the prostate. As a comparison, the ROC curve analysis of blood PSA levels was also determined (AUC: 0,893, *p* < 0,001), resulting in a sensitivity of 91,7% and specificity of 60% when used as a classifier for PCa in our study. To determine if a multiplex model could improve performance over single biomarkers for discriminating PCa from non-malignant samples, the three previously significant miRNAs were analysed in a multivariate logistic regression analysis. Interestingly, this analysis resulted in a model that included the miR-142-3p and miR-142-5p expression values (AUC: 0,728, *p* = 0,018). In this case, the sensitivity and specificity for predicting the PCa samples were 83,3% and 60% respectively. Strikingly, when compared with PSA, a moderate increased value of specificity (Sn: 91,7 and Sp: 73,3%) was obtained when PSA + miR-142-3p + miR-142-5p were included in the model (AUC: 0,911, *p* < 0,001) (Fig. 4A).

Additionally, the same analysis was performed in samples from individuals who presented PSA levels ≥4 ng/ml in order to discriminate PCa from BPH individuals. In this case blood PSA levels resulted in a high sensitivity of 100% but in a low specificity of 14,3% for PCa as previously described (AUC: 0,771, *p* = 0,032), the same as the ones obtained when the three miRNAs (miR-142-3p + miR-142-5p + miR-223-3p) were included in the model (AUC: 0,673, *p* = 0,171). When all four variables (PSA + miR-142-3p + miR-142-5p + miR-223-3p) were introduced in the analysis it resulted in a model with higher accuracy (AUC: 0,821, *p* = 0,011) and a better use for diagnosis: sensitivity of 91,7% and specificity of 42,9% (Fig. 4B).

**Figure 5:**
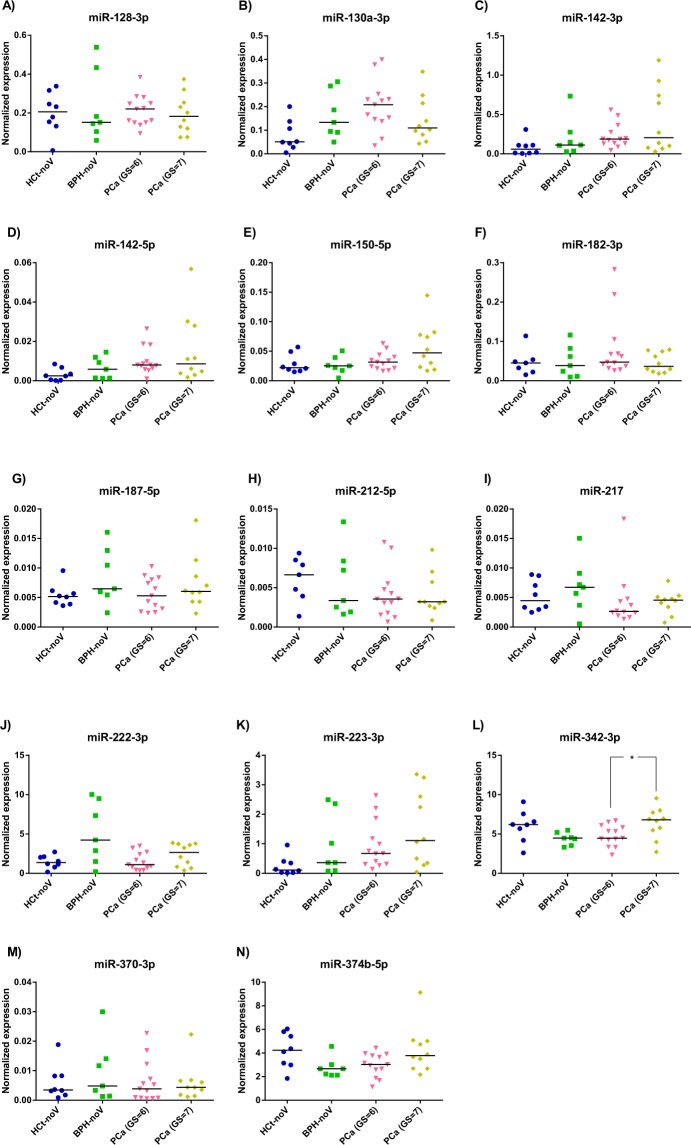
SP exosome miRNA levels in PCa samples with different severities of tumour defined by Gleason Score. Expression profiling, at the testing/validating stage, of the miRNAs in exosomes from semen of healthy controls (HCt), benign prostate hyperplasia-non vasectomised (BPH-noV), Gleason 6 classified prostate cancer [PCa (GS = 6)] and Gleason 7 classified prostate cancer [PCa (GS = 7)]. The horizontal bar displays the median cellular expression level. Significant differences between groups are indicated: *p < 0,05; **p < 0,01 (Mann Whitney U test)

**Figure 6:**
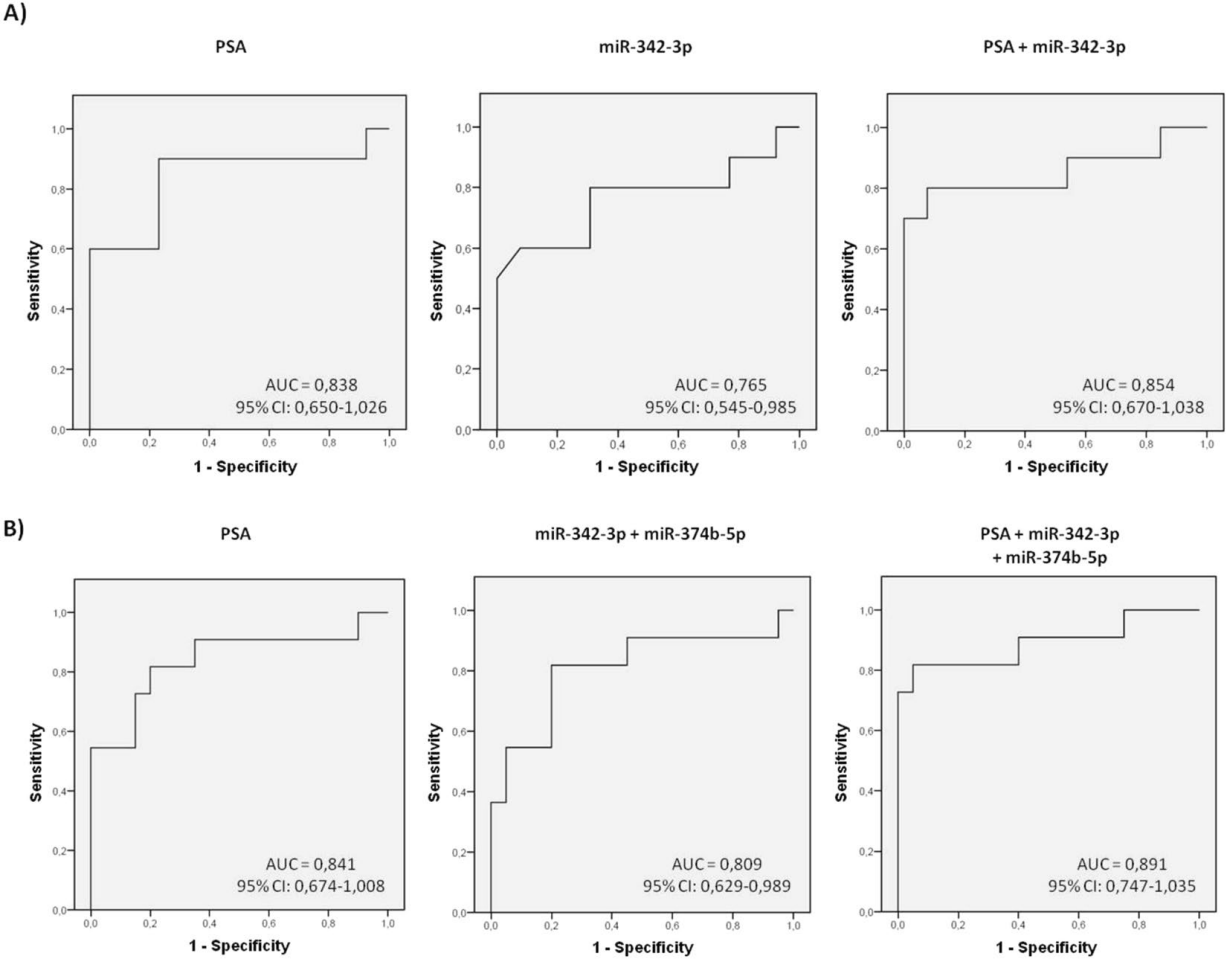
MiRNA-based models as prognostic classifiers. Receiver operating characteristic (ROC) curves showing the predictive efficiency for distinguishing A) Gleason 6 (GS6) from Gleason 7 (GS7) PCa samples and B) (BPH + GS6) samples from GS ≥ 7 PCa samples, by using serum PSA and compared with the combined model [either PSA + miR-342-3p for A) and PSA + miR-342-3p + miR-374b-5p for B)] obtained at the testing/validation stage. The multivariate models were obtained by performing a multivariate binary logistic regression analysis (backward stepwise, conditional method). AUC: area under the curve; 95% CI: a 95% of confidence interval.

Furthermore, the same type of analysis was performed in order to determine if a multiplex miRNA model could reflect the severity or degree of PCa affectation. We found miR-342-3p can distinguish (AUC: 0,765; *p* = 0,032) between PCa samples with GS6 and those with GS7 in the biopsy (Fig. 5). Again, an increased value of true positive and negative rates for predicting a higher PCa Gleason score (60 and 100% respectively; AUC 0,854, *p* = 0,004) was obtained when miR-342-3p + PSA was included in the model, much better than the ones obtained using single biomarkers: PSA (60 and 84.6%; AUC 0,838, *p* = 0,006) or miR-342-3p (60 and 76,9%) (Fig. 6A).

What is more, miR-342-3p (AUC: 0,800; *p* = 0,006; Sn: 63,6%; Sp: 90%), miR-374b-5p (AUC: 0,768; *p* = 0,015; Sn: 45,5%; Sp: 90%) and the combination of both miRNAs (AUC: 0,809; *p* = 0,005; Sn: 54,5%; Sp: 80%) permit the discrimination between a group of men with GS ≥ 7 and a group of men presenting PSA levels > 4 ng/ml without cancer or with GS6. These results are similar to the results obtained for PSA (AUC: 0,841, *p* = 0,002; Sn: 54,5%; Sp: 90%), indicating that these markers may have prognostic potential. The combined model (PSA + miR-342-3p + miR-374b-5p) resulted in a higher predictive accuracy (AUC: 0,891; *p* < 0,001) with a clinically useful sensitivity and specificity (81,8% and 95% respectively) (Fig. 6B).

### Potential target genes altered by the identified miRNAs

Identifying the target genes of the putative miRNA biomarkers is important for understanding their role in the aetiology of the disease. The *in silico* analysis by using the miRNet web-based platform allow us to generate a list of target genes and candidate pathways for the miRNAs in exosomes (Supplementary Tables S3 and S4).

The analysis resulted in a list of 1251 target genes (Supplementary Table S3) for the miRNAs involved in 186 KEGG (Kyoto Encyclopedia of Genes and Genomes) pathways (Supplementary Table S4). Interestingly, prostate cancer signalling was among the five first positions of canonical pathways consisting of 20 deregulated target genes (Table 4).

**Table 4:** Potential target genes altered by the identified SP exosomal miRNAs involved in prostate cancer signalling.

miRNA	Target Gene name	Molecular function	miRTarBase	Validation methods
hsa-miR-142-3p	*CREB3L2*	Transcriptional activator; DNA-binding transcription factor, RNA polymerase II-specific	MIRT440881	NGS
hsa-miR-142-3p	*CTNNB1*	Cell adhesion, cell division	MIRT524299	NGS
hsa-miR-142-3p	*PIK3CG*	Involved in immune response	MIRT439973	NGS
hsa-miR-142-3p	*CDKN1B*	Cell cycle progression control	MIRT494317	NGS
hsa-miR-142-5p	*PTEN*	Tumor suppressor, focal adhesion	MIRT732387	Reporter assay, WB, qPCR
hsa-miR-142-5p	*MAP2K1*	Cell proliferation, differentiation, transcription regulation and development	MIRT281791	NGS
hsa-miR-142-5p	*MAPK1*	Cell proliferation, differentiation, transcription regulation and development	MIRT506656	NGS
hsa-miR-142-5p/hsa-miR-374b-5p	*CCND1*	Cell cycle progression control	MIRT060553/MIRT504733	NGS
hsa-miR-374b-5p	*GSK3B*	Energy metabolism, inflammation, ER-stress, mitochondrial dysfunction, and apoptotic pathways	MIRT504733	NGS
hsa-miR-374b-5p	*AKT1*	Inactivates components of the apoptotic machinery	MIRT735321	Reporter assay, WB, qPCR, microarray
hsa-miR-223-3p	*TP53*	Tumor suppressor	MIRT465435	NGS
hsa-miR-223-3p	*HSP90B1*	Chaperone with roles in stabilizing and folding other proteins.	MIRT007086	Reporter assay, WB
hsa-miR-223-3p	*CDK2*	Cell cycle regulation	MIRT053070	Reporter assay, WB, qPCR
hsa-miR-223-3p	*FOXO1*	Myogenic growth and differentiation	MIRT006733	Reporter assay, WB, qPCR
hsa-miR-223-3p	*CHUK*	A component of a cytokine-activated protein complex that is an inhibitor of the transcription factor NF-kappa-B complex	MIRT005555	Reporter assay, WB, qPCR
hsa-miR-223-3p	*MDM2*	Ubiquitin ligase that promotes tumor formation by targeting tumor suppressor proteins	MIRT486453	NGS
hsa-miR-223-3p/hsa-miR-342-3p	*IGF1R*	Has tyrosine kinase activity and plays a critical role in transformation events.	MIRT006244/MIRT623633	Reporter assay, WB, qPCR, microarray/NGS
hsa-miR-342-3p	*E2F1*	Control of cell cycle and action of tumor suppressor proteins	MIRT734732	Reporter assay, WB, qPCR
hsa-miR-342-3p	*ATF4*	Transcription factor	MIRT043684	NGS
hsa-miR-342-3p	*IKBKG*	Activates NF-kappaB resulting in activation of genes involved in inflammation, immunity, cell survival, and other pathways	MIRT732518	Reporter assay, WB

## Discussion

The prediction of PCa in the early stage of the disease is one of the most important objectives in male urology. A significant decrease in deaths due to PCa has been associated with the use of serum PSA test for nearly 30 years. However, considerable controversy has been raised over its value after recognizing that PSA testing has caused over diagnosis and over treatment of PCa. This has generated increased efforts to identify diagnostic/prognostic biomarkers to efficiently discriminate between PCa tumours that need treatment and clinically insignificant tumours or benign prostatic diseases that do not require intervention but should undergo active surveillance.

Aberrant miRNA expression has been previously linked to cancer in prostate cells^25^. MiRNAs in biofluids have emerged as potentially useful biomarkers for a variety of conditions such as cancer. Several miRNAs, isolated from blood and urine, have previously been shown to be dysregulated in patients with PCa compared with normal control patients (reviewed in^26^) with divergent results. Although semen is an ideal biofluid for biomarker detection and has proved useful for detecting diseases affecting organs of the male reproductive system, only a few studies to date have analysed the expression level of miRNAs in the semen, and specifically in the non-sperm cellular fraction of seminal fluid, for the detection of PCa^6,27^. However, the aim of our study was to determine if a miRNA signature from the exosome-associated fraction of semen would help to identify patients with cancer, as the exosome cargo closely reflects the donor cell cargo. We used the same approach in male infertility for predicting the origin of azoospermia and the presence of sperm in the testis^24^. For prostate cancer, exosomes secreted by the prostate can be isolated from prostatic secretions, and thus from semen. MiRNAs are protected by the exosomal membrane from being degraded which makes exosomal miRNAs ideal biomarkers for tumour diagnosis. To our knowledge, this is the first time that miRNA expression profile from semen exosomes has been studied in order to establish a potential diagnostic semen miRNA panel to reduce the need for prostate biopsies in patients with moderately elevated PSA levels, many of whom ultimately will not have PCa.

Firstly, in the present study we focused our attention on those miRNAs that were statistically dysregulated in PCa and/or BPH patients, when compared to healthy controls. The presence of vasectomy was taken into account for the interpretation of the results of this study, as the number of couples selecting male vasectomy as a contraceptive method has been increasing in recent years. The practice of vasectomy affects the concentration of certain exosomal miRNAs in SP because the fluid from testis and epidydimis cannot reach semen, thus miRNAs that were highly under-expressed ( > 80%) in vasectomised samples were avoided for further analysis. After that, we found that the levels of 50 miRNAs in exosomes were altered in PCa and BPH compared with healthy controls when assessed by miRNA qPCR arrays that, profiled 634 human miRNAs. These results were validated for 14 miRNAs in a larger cohort of patients and specifically three of them (miR-142-3p, miR-142-5p, miR-223-3p) were confirmed as being at significantly higher levels in men with moderately elevated PSA levels and biopsy-proven cancer. The corresponding up-regulation of these three miRNAs in PCa and BPH tissue suggest that the concentration of these miRNAs in SP exosomes can indeed reflect prostate health. Interestingly, these three miRNAs are over-expressed in malignant tumors^26^ and specifically, miR-223-3p up-regulation has been previously linked to PCa^24,25^.

Additionally, our results suggest that miRNAs in exosomes from seminal fluid are useful as biomarkers for PCa when combined with PSA data. Firstly, to discriminate PCa from (HCt + BPH) control individuals, PSA + miR-142-3p + miR-142-5p were included in the model (AUC: 0,914, *p < *0,001) resulting in higher values for sensitivity and specificity (91,7 and 73,3%) and secondly, a model combining four variables (PSA + miR-142-3p + miR-142-5p + miR-223-3p) was useful to discriminate PCa from BPH individuals with high accuracy (AUC: 0,821, *p* = 0,011) and would be more suitable for clinical diagnosis: sensitivity of 91,7% and specificity of 42,9%, increasing PCa specificity of the PSA screening test. Said with other words, with the latter combined model, 3 unnecessary biopsies would be saved for every 10 patients (improving the specificity of the test from 14% to 43%, corresponding to BPH individuals correctly identified) at the expense of losing one true positive result (from 100% to 91,7%) that can be rescued in the future thanks to the active surveillance of the patients.

The prognostic predictive accuracy of (PSA + miR-342-3p + miR-374b-5p) panel to discriminate between a group of men with GS ≥ 7 and a group of men presenting moderate PSA levels without cancer or with GS6, is also shown and indicates that it has a clinically useful prognostic potential (Sn: 81,8% and Sp: 95%). Again, the miRNA-based classifier is superior to PSA alone for PCa prognostics. In this case, 28% more patients with GS7 would also be identified with the combined model.

The *in silico* identification of 20 miRNA target genes involved in PCa signalling pathway indicate that their regulation by miRNAs may be crucial in PCa pathogenesis. MiRNAs can function as tumour suppressors and/or oncogenes, depending on the cellular function of their targets. Previous results from other laboratories agreed with the role of these dysregulated exosome-contained SP miRNAs in the aetiology of PCa disease. This is the case of miR-223-3p that was found up-regulated in PCa cell lines (C4-2, LNCaP, PC3, DU-145) and PCa tissues^28,29^, and to negatively regulate *FOXO3* and *SEPT6* leading to a decrease in cell death.

Similarly, as we found in semen from PCa patients, miR-142-3p/5p is up-regulated in lung adenocarcinoma and breast cancer (reviewed in^30^). What is also emerging is that each isoform (miR-142-3p vs. -5p) can have overlapping as well as specific transcriptional targets, and in some cases it can have opposite effects on specific pathways. High levels of miR-142-5p expression correlate with cancer progression through regulating the TGFß-signalling pathway. In particular, miR-142-5p targets *Smad3* thereby suppressing TGFß-induced growth inhibition in cancer cells^31^. As examples, miR-142-5p has an oncogenic role in colorectal cancer through the modulation of tumour suppressor *KLF6* expression^32^; miR-142-5p promoted the proliferation and migration of renal cell carcinoma cells by targeting *BTG3*^33^. On the other hand, a study performed by Isobe and colleagues demonstrated that miR-142-3p triggers the progression of tumours in human breast stem cells by targeting *APC*. Loss of APC expression activates the canonical WNT/CTNNB1 signalling pathway leading to excessive cell growth in breast tissue^34^.

Also in line with our results, miR-342-3p and miR-374b-5p were described to be associated with tumour aggressiveness. Specifically, miR-342-3p was found to be over-expressed in colon cancer; this over-expression was associated with lower survival time^35^ although in other types of tumours, such as liver and lung cancers, it is described as acting as a tumour suppressor^36,37^. This seeming inconsistency may be explained by cell type-specific differences as mentioned above. Referring to miR-374b-5p, it contributes to gastric cancer cell metastasis and invasion^38^.

Profiling of circulating miRNA in PCa patients has been previously carried out in serum and urine, in both total fluid and in exosomes, although the altered pattern of miRNA expression was different to the one we found in semen exosomes. This could be partially a consequence of the proportion of prostate-miRNA in the fluid composition determined by its origin; for example, prostatic secretions constitute 40% of semen, quite different to the proportion found in serum, although it has been experimentally demonstrated that increased tissue expression of miRNAs leads to elevated levels in serum. Additionally, semen represents a liquid biopsy from the whole prostate gland, whereas the prostate fluid in urine after prostate massage is derived from the posterior part of the gland and thus malignancies from the anterior part of the prostate might be missing. What is more, it should be noted that most PCa studies in serum and urine involve both localised/locally advanced and metastatic disease in contrast to our study which concerns PCa in the early stages, so it could therefore result in additional biomarkers.

In conclusion, we demonstrated that clinically relevant, quantitative changes in the transcript levels of miRNAs can be detected in the semen exosomes of reproductive cancer patients. In our study we showed a miRNA signature from the exosome-associated fraction of semen as a molecular classifier that may improve the efficiency of detection of PCa and of determining the severity/aggressiveness of PCa at the time of diagnosis. Our results also showed that the over-expression of these miRNAs in semen perfectly fits with their abundance in prostate and, therefore they are able to reflect the prostate health. For their diagnostic use, we propose the analysis of miR-142-3p, miR-142-5p and miR-223-3p in combination with blood PSA concentration to distinguish benign from malignant tumours. These biomarkers are able to reduce unnecessary biopsies in the PSA “grey zone” by over 30%. Additionally, a combined model of miR-342-3p, miR-374b-5p and PSA levels was also associated with the Gleason Score and therefore may have a prognostic value. The inclusion of miRNA levels and PSA levels in the model predicts PCa and its severity better than is commonly reported in PSA screening. As the next step, further prospective studies on larger cohorts of patients involving different participants are required to confirm/validate our findings on the diagnostic and/or prognostic role of such miRNA panel before it could be adopted into clinical practice. The screening of miRNAs in seminal plasma of patients with prostate cancer may provide additional information on the aberrant signalling pathway which could potentially be blocked by a targeted therapy. The clinical use of these non-invasive biomarkers could, therefore, contribute to an understanding of the molecular mechanisms underlying prostate cancer initiation and progression. Taking all this together, our findings contribute to the search for the most valuable genetic markers as diagnostic and prognostic tools that are useful in the clinical setting and that would improve diagnosis and avoid unnecessary biopsies with the potential to reduce cost of the system and reduce complication rates due to prostate biopsies and thus improving patient outcome. Future studies should investigate semen miRNAs as biomarkers in other clinically relevant stages of PCa progression, such as prediction of metastasis, survival and/or response to treatment.

## Material and Methods

### Subjects of study

Semen specimens were collected from 11 healthy individuals consulting for vasectomy (control group 1: HCt) at the Andrology Service of Fundació Puigvert and 31 individuals consulting for diagnosis of PCa who underwent routine prostate screening including PSA testing and DRE at the Urology Service of the Bellvitge Hospital. Patients that presented moderate PSA levels (4-18 ng/ml) were selected with consent to undergo prostate biopsy. The latter group comprised: 24 men with biopsy-proven PCa including men who were previously successfully vasectomised (PCa-V, n = 8) and non-vasectomised individuals (PCa-noV, n = 16); and additionally, 7 non-vasectomised individuals with benign prostatic hyperplasia (BPH) or prostate enlargement (control group 2) who presented elevated PSA levels (>4 ng/ml) but no detectable cancer on biopsy (Table 1; Supplementary Table S1).

The study was approved by the Ethical Review Board of both institutions. The participants signed an informed consent form. All methods were performed in accordance with the relevant guidelines and regulations. The research is conceived as a pilot study for the assessment of semen exosome miRNA-based biomarker test useful for PCa diagnosis, which involves the selection of the miRNAs to be included in the test. Thus, a two-stage (miRNA screening and miRNA testing/validating stage) case control study was designed to analyse the exosome miRNA levels in semen. The selected miRNAs were subsequently used for the development of combined miRNA-based diagnostic classifiers for PCa (Fig. 1).

### Semen samples and exosome isolation

Semen samples were obtained by masturbation after 3–5 days of sexual abstinence. They were allowed to liquefy for 30 min at 37 °C. Isolation of exosomal vesicles was performed by differential centrifugation steps (1600 xg for 10 min, then 16000 xg for 10 min at 4 °C) including one microfiltration step (0,22 μm pore size) and ultracentrifugation (100000 xg for 2 h at 4 °C) to sediment exosomes as described elsewhere^13,24^. Nanoparticle tracking analysis was performed by NanoSight NS300 (Malvern Instruments Ltd; UK) showing that >90% of the recovered particles had a size of <200 nm (Supplementary Table S5). Slightly larger vesicles were found in PCa when compared to control samples, leading to significant variations in the vesicle distribution between HCt and PCa groups, considering vesicle size mean (*p* = 0,048), mode (*p* = 0,024) and D50 (*p* = 0,024) values.

### Small RNA-containing total RNA isolation

#### From semen exosomes

Total RNA was isolated as previously described^24^. Briefly, with the purpose of degrading the residual RNA outside the vesicles, the exosome suspension was treated with RNAse A (Qiagen NV; Germany) (100 μg/ml final reaction concentration; 15 min at 37 °C). Total RNA was obtained from exosomes using the miRCURY RNA Isolation Kit-Cell and Plant (Exiqon; Denmark). RNA concentration was calculated by using the QUBIT fluorometer and the Quant-iT RNA Assay kit (Invitrogen; California, USA). All RNA samples presented an OD 260/280 nm ratio ≥ 1,7 when using a Nanodrop UV-Vis spectrophotometer (Thermo Fisher Scientific; Massachusetts, USA).

#### From tissue

Tissue small RNA-containing total RNA was additionally obtained with a mirVana miRNA Isolation Kit (Ambion) from frozen biopsies (-80 °C) of different organs of the reproductive tract. Specifically, testicular biopsies (n = 2) were previously obtained from infertile men due to an obstruction of the ductal system consulting for reproduction assisted treatment that includes sperm retrieval and cryopreservation purposes^39^. Those individuals have a conserved spermatogenesis testicular pattern. Post-mortem epididymis (n = 2) and prostate (n = 2) samples as well as prostate samples from PCa (n = 2) and BPH (n = 2) patients were kindly ceded by the Pathological Anatomy Service of Bellvitge Hospital.

The RNA quality assessment was performed using the Agilent 2100 Bioanalyzer (Agilent Technologies, Waldbronn, Germany). Tissular RNA samples included in the study had a 28 S/18 S ratio > 1.4 and a RIN value > 7.5.

### Exosomal miRNA quantitative real-time PCR profiling

It was essentially performed as previously described^24^. For the miRNA screening, RNA was reverse transcribed (RT) using the miRCURY LNA™ Universal RT microRNA PCR, Polyadenylation and cDNA synthesis kit (Exiqon; Denmark). cDNA was diluted 50x and assayed in 10 µl PCR reactions according to the protocol for miRCURY LNA™ Universal RT microRNA PCR. Each miRNA was assayed once by quantitative real-time PCR (qPCR) using ExiLENT SYBR Green mastermix on the microRNA Ready-to-Use PCR Human panels I and II that include 634 mature miRNAs of miRBase (www.mirbase.org/) in a LightCycler® 480 Instrument (Roche; Switzerland). Experiments were conducted at Qiagen Genomic Services (Germany). Details and conditions of analysis of the amplification curves and efficiencies are described elsewhere^24,40^.

Next, to correct for potential overall differences in amount and quality between the samples, the raw data (Crossing Points Cp values) were normalised for each sample to the mean of the 50 most stable assays (mean 50) that were detected in all samples: dCp = mean 50 Cp – assay Cp. Those assays were previously selected as being the ones with the lowest coefficient of variation (CV < 0,017) of Cp values among samples in the study (Supplementary Table S6) as well as showing no statistical differences in absolute expression levels between groups, either individually or for the mean value.

The relative quantitative method of 2^dCp^ was used to calculate the relative quantification (RQ) miRNA expression values.

### Validation of miRNA candidates by RT-qPCR analysis

The procedure was similar to that previously used^24^. First-stranded cDNA specific for miRNA was obtained by RT of 50 ng of exosomal RNA in 10 µl, using the Universal cDNA synthesis kit II (Exiqon; Denmark). For qPCR analysis, cDNA was diluted (12 × ) and assayed in 10 µl PCR reactions containing ExiLENT SYBR Green master mix (Exiqon; Denmark). Duplicate amplification reactions of individual assays (LNA™-enhanced miRNA qPCR primers; Supplementary Table S7) were carried out on a Lightcycler® 96 Instrument (Roche; Switzerland). Target miRNA expression for exosomes in semen samples was calculated relative to the mean expression value of miR-30e-3p and miR-126-3p, chosen from the exosomal miRNA qPCR profiling study as being among the most stable miRNAs (Supplementary Table S6). The RQ values were calculated using the 2^dCp^ strategy. The same procedure was applied to determine tissue expression profiling of miRNA candidates with some modifications: 20 ng of tissue RNA were reverse transcribed and cDNA was diluted 80 × .

### Determining *in silico* target genes of miRNAs

We used the miRNet web-based platform^41^ to identify target genes and pathways potentially altered by the miRNA signature. The miRNet predicted targets are determined experimentally as part of larger experiments such as microarray, RNA-seq, qPCR and PAR-CLIP, and immunoprecipitation method for identifying the binding sites of cellular RNA-binding proteins (RBPs) and miRNA-containg ribonucleoportein complexes (miRNPs) and each identified target gene is supported by the experiment name and PubMed literature^41^. Additional analysis of the miRNA expression in the TCGA dataset and functional analysis was performed by using this platform^41^.

### Statistical analysis

The non-parametric Kruskal–Wallis test was used to analyze the differences in clinical data and absolute expression levels of reference genes. Unpaired two-tailed Student’s t test was used to analyze the differences in relative expression of miRNAs between groups in the miRNA profiling study and the Benjamini-Hochberg procedure for multiple testing corrections, using a maximum discovery rate of 5%, was applied to calculate the False Discovery Rate (FDR) for each of the *p*-values obtained. The non-parametric Mann–Whitney U-test was used to evaluate differences in relative expression of selected miRNAs between groups. Pearson product-moment correlation coefficients (*r*) were calculated to determine the correlation between the miRNA RQ values and the various parameters in semen analysis, blood analysis or prostate biopsy in patient groups and controls. Receiver operating characteristic (ROC) curve analysis^42^ of the RQ values was used to distinguish the samples showing malignancy of prostate tumour. Accuracy was measured as the area under the ROC curve (AUC)^42^. The threshold value was determined by Youden’s index, calculated as sensitivity plus specificity-1^43^. A multivariate binary logistic regression analysis was used for selection of the optimal combination of variables associated with the presence of PCa or with the aggressiveness of the disease. A backward stepwise (conditional) method was used to drop insignificant terms. The binary logistic regression model provides the following estimation of the logit function:

$${\rm{Logit}}(p)={\rm{B}}0+{\rm{B}}1{\rm{X}}1+{\rm{B}}2{\rm{X}}2+\ldots $$

Where p = P (presence of prostate cancer), Logit (p) = log(p/(1-p)) = log(odds), B = logOR and Xn = the expression values of the miRNAs. Therefore, if we use this estimated model as a prediction model, with the standard classification cutoff of 0,5, we would classify individuals with a positive Logit function estimation as “positive for PCa” and individuals with negative Logit function estimation as “negative for PCa”.

Statistical analyses were performed using the SPSS software version 15.0 (SPSS Inc.; IBM; IL, USA). A value of *p* ≤ 0,05 was considered significant.

## Supplementary information

Supplementary Information
