# Supplementary material for: Semen miRNAs Contained in Exosomes as Non-Invasive Biomarkers for Prostate Cancer Diagnosis
Source: Sci Rep. 2019 Sep 24;9:13772. doi: 10.1038/s41598-019-50172-6 (PMC6760223; doi:10.1038/s41598-019-50172-6)
Supplement: Supplementary file 1 — Supplementary Information [file 41598_2019_50172_MOESM1_ESM.pdf]

SUPPLEMENTARY INFORMATION

SEMEN miRNAs CONTAINED IN EXOSOMES AS NON-INVASIVE BIOMARKERS FOR PROSTATE CANCER DIAGNOSIS

MARIA BARCELÓ[1], MANEL CASTELLS[2], LLUÍS BASSAS[3], FRANCESC VIGUÉS[2], SARA LARRIBA[1*]

[1]Human Molecular Genetics Group- Bellvitge Biomedical Research Institute (IDIBELL), 08908 Hospitalet de Llobregat, Barcelona, Spain

[2]Urology Service, Bellvitge University Hospital-ICS, 08908 Hospitalet de Llobregat, Barcelona, Spain

[3]Laboratory of Seminology and Embryology, Andrology Service-Fundació Puigvert, 08025 Barcelona, Spain

***Correspondence address**

Tel. +34 93 260 74 25 (ext. 73 38); Fax. +34 93 260 74 14; e-mail: slarriba@idibell.cat

Running title

Aberrant miRNA content of seminal plasma exosomes in malignant prostate cancer

A)

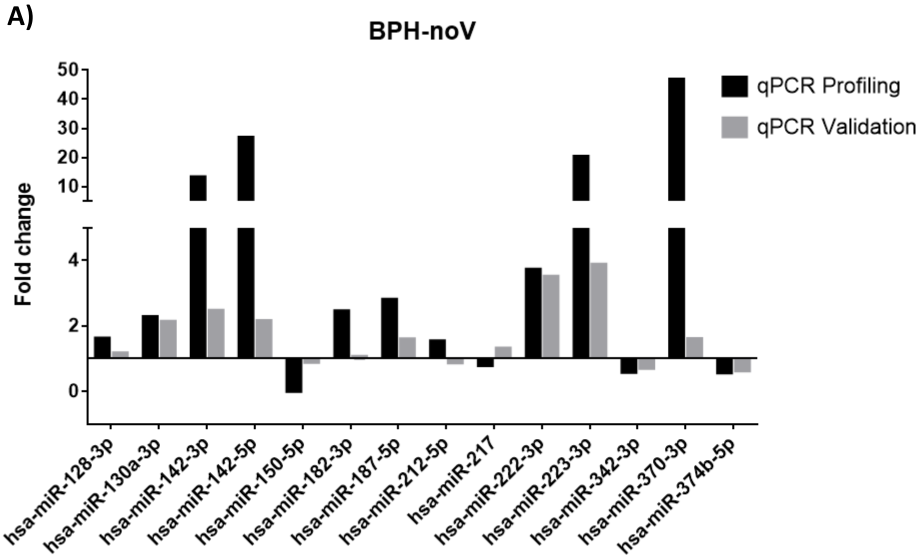
BPH-noV
qPCR Profiling
qPCR Validation
Fold change
50
40
30
20
10
4
2
0
hsa-miR-128-3p
hsa-miR-130a-3p
hsa-miR-142-3p
hsa-miR-142-5p
hsa-miR-150-5p
hsa-miR-182-3p
hsa-miR-187-5p
hsa-miR-212-5p
hsa-miR-217
hsa-miR-222-3p
hsa-miR-223-3p
hsa-miR-342-3p
hsa-miR-370-3p
hsa-miR-374b-5p

B)

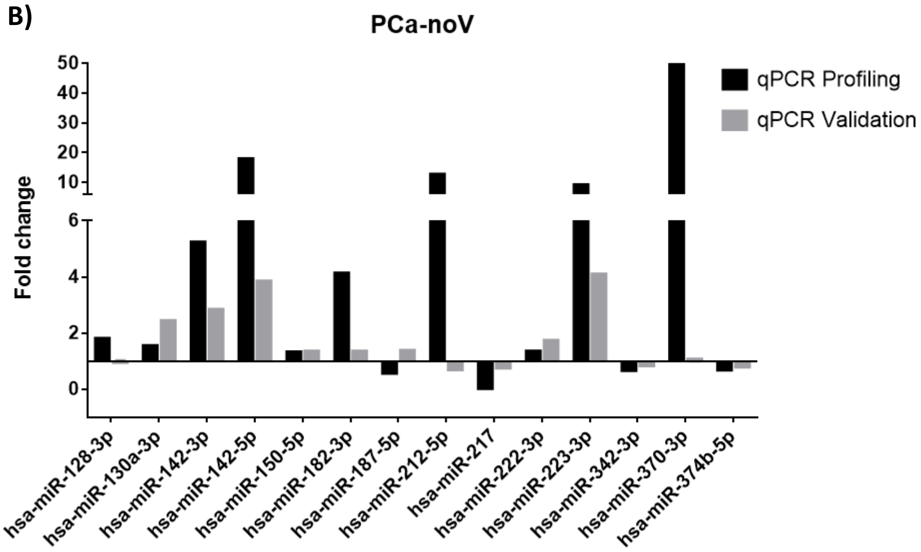
PCa-noV
qPCR Profiling
qPCR Validation
Fold change
50
40
30
20
10
6
4
2
0
hsa-miR-128-3p
hsa-miR-130a-3p
hsa-miR-142-3p
hsa-miR-142-5p
hsa-miR-150-5p
hsa-miR-182-3p
hsa-miR-187-5p
hsa-miR-212-5p
hsa-miR-217
hsa-miR-222-3p
hsa-miR-223-3p
hsa-miR-342-3p
hsa-miR-370-3p
hsa-miR-374b-5p

C)

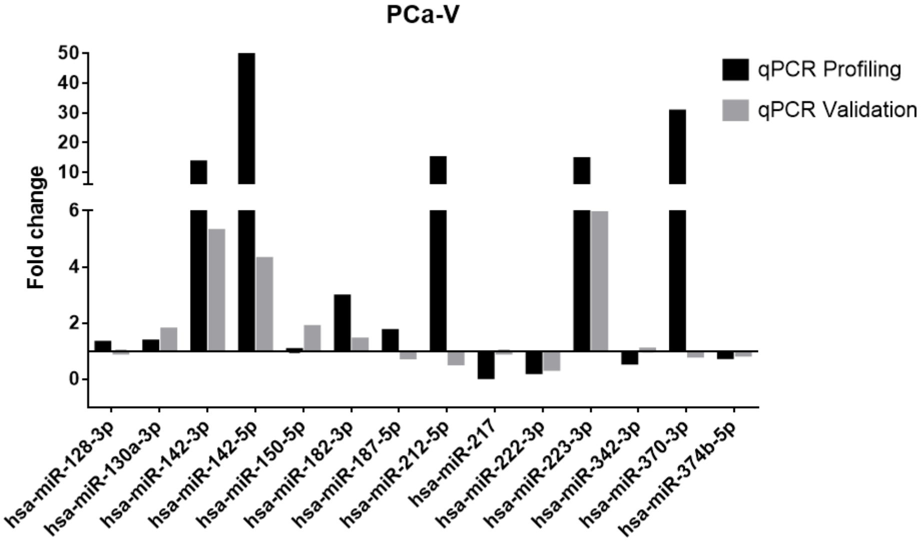
PCa-V
qPCR Profiling
qPCR Validation
Fold change
50
40
30
20
10
6
4
2
0
hsa-miR-128-3p
hsa-miR-130a-3p
hsa-miR-142-3p
hsa-miR-142-5p
hsa-miR-150-5p
hsa-miR-182-3p
hsa-miR-187-5p
hsa-miR-212-5p
hsa-miR-217
hsa-miR-222-3p
hsa-miR-223-3p
hsa-miR-342-3p
hsa-miR-370-3p
hsa-miR-374b-5p

Supplementary Figure S1 Exosomal miRNA abundance comparison between the RT-qPCR panels (miRNA screening) and the RT-qPCR individual assays (miRNA validation). The mean fold-changes of A) BPH-noV, B) PCa-noV and C) PCa-V compared to HCt controls are shown.

Supplementary Table S1

Clinical data of individuals included in the study of miRNA content of seminal plasma exosomes in PCa.

Patient No.	Subgroups	Age (years)	Vasectomized?	PSA ng/ml (pre-biopsy)	Gleason score (biopsy) GS-B	Clinical stage (cT+N+M)	Treatment	Gleason score (surgery) GS-S	Pathologic stage (pT+N)
1	HCt	39	no	nd	--	--	--	--	--
2	HCt	39	no	nd	--	--	--	--	--
3	HCt	40	no	nd	--	--	--	--	--
4	HCt	40	no	nd	--	--	--	--	--
5	HCt	37	no	nd	--	--	--	--	--
6	HCt	42	no	nd	--	--	--	--	--
7	HCt	45	no	nd	--	--	--	--	--
8	HCt	46	no	nd	--	--	--	--	--
9	HCt	41	no	nd	--	--	--	--	--
10	HCt	44	no	nd	--	--	--	--	--
11	HCt	35	no	0,62	--	--	--	--	--
12	BPH/HGPIN	53	no	5,93	--	--	--	--	--
13	BPH	67	no	4,69	--	--	--	--	--
14	BPH	64	no	4,59	--	--	--	--	--
15	BPH	61	no	4,97	--	--	--	--	--
16	BPH	59	no	2,30	--	--	--	--	--
17	BPH	59	no	6,07	--	--	--	--	--
18	BPH	56	no	4,68	--	--	--	--	--
19	PCa-noV	53	no	9,40	6(3+3)	cT1c_N0_MX	RP	6(3+3)	pT2c_NX
20	PCa-noV	50	no	4,50	6(3+3)	cT1c_N0_MX	RP	7(3+4)	pT2c_NX
21	PCa-noV	61	no	10	6(3+3)	cT1c_N0_MX	RP	6(3+3)	pT2c_NX
22	PCa-noV	68	no	5,75	8(4+4)	cT1c_N0_M0	RP	8(4+4)	pT2a_N0
23	PCa-noV	59	no	6,85	6(3+3)	cT1c_N0_MX	RP	7(4+3)	pT2c_NX
24	PCa-noV	58	no	5,97	6(3+3)	cT1c_NX_MX	RP	7(3+4)	pT2c_NX
25	PCa-noV	59	no	4,25	7(3+4)	cT1c_N0_MX	RP	7(3+4)	pT2c_NX
26	PCa-noV	59	no	6,80	7(3+4)	cT1c_NX_MX	RP	7(3+4)	pT2c_NX
27	PCa-noV	61	no	12,40	7(3+4)	cT3a_N0_M0	RP	7(3+4)	pT2c_NX
28	PCa-noV	59	no	4,97	6(3+3)	cT1c_NX_MX	RP	6(3+3)	pT2c_NX
29	PCa-noV	56	no	5,99	7(4+3)	cT1c_NX_MX	RP	7(4+3)	pT3a_NX
30	PCa-noV	67	no	10,46	7(3+4)	cT2c_N0_MX	AS	nd	nd
31	PCa-noV	53	no	5,03	6(3+3)	cT1c_NX_MX	RP	6(3+3)	pT2c_NX
32	PCa-noV	62	no	5,10	6(3+3)	cT2c_N0_MX	AS	nd	nd
33	PCa-noV	63	no	6,28	7(4+3)	cT2c_N0_MX	RP	7(4+3)	pT3a_NX
34	PCa-noV	54	no	17,70	7(4+3)	cT3a_N0_M0	RP+LDN	7(4+3)	pT3a_N0
35	PCa-V	43	yes	11,90	7(3+4)	cT2c_N0_MX	RP	7(3+4)	pT2c_NX
36	PCa-V	55	yes	17	7(3+4)	cT2c_N0_MX	RP+ LDN	7(3+4)	pT2c_N0
37	PCa-V	64	yes	4,24	6(3+3)	cT1c_N0_MX	AS	nd	nd
38	PCa-V	67	yes	5,86	6(3+3)	cT2c_N0_MX	AS	nd	nd
39	PCa-V	56	yes	12,51	7(3+4)	cT3a_N0_M0	RP + LDN	7(3+4)	pT3a_N0
40	PCa-V	50	yes	4,90	6(3+3)	cT1c_NX_MX	RP	6(3+3)	pT2c_NX
41	PCa-V	67	yes	5,41	6(3+3)	cT2a_N0_MX	AS	nd	nd
42	PCa-V	67	yes	4,96	6(3+3)	cT1c_NX_MX	AS	nd	nd

HCt: healthy control; BPH: benign prostate hyperplasia; HGPIN: high-grade prostatic intraepithelial neoplasia; PCa-noV: prostate cancer in a non-vasectomized individual; PCa-V: prostate cancer in a vasectomized individual; PSA: prostate-specific antigen; T: primary tumor; N: nodal status; M: distal metastasis; RP: radical prostatectomy; AS: active surveillance; LDN : lymphadenectomy

Samples depicted in blue refer to those samples included in miRNA profiling phase of the study.

Supplementary Table S2. Summary of miRNA expression data in PCa and in BPH phenotypes related to HCt

Statistically increased miRNA expression levels are depicted in green (colour intensity is inversely related to the expression value); statistically decreased miRNA expression levels are depicted in red (colour intensity is related to the expression value), when compared with controls

Cp values >38 are depicted in bold.

* p≤0,05; ** p≤0,005. Statistically significant p-values are depicted in bold.

miRNAs which fullfilled the criteria as candidate biomarkers are highlighted in brown; those selected for validation are depicted in italics

					miRNA expression				***p* -value**		
miRNA	Average *Cp* $_{PCa\_V}$	Average *Cp* $_{PCa\_noV}$	Average *Cp* $_{BPH}$	Average *Cp* $_{HCt}$	PCa_V	PCa_noV	BPH	HCt	PCa_V - HCt	PCa_noV - HCt	BPH - HCt
**hsa-let-7a-5p**	22,16	21,40	22,05	21,21	0,89	1,01	0,84	1,00	> 0,10	> 0,10	> 0,10
**hsa-let-7b-3p**	31,74	31,02	31,64	30,73	0,85	0,95	0,80	1,00	> 0,10	> 0,10	> 0,10
**hsa-let-7b-5p**	23,13	22,42	22,88	22,39	1,02	1,13	1,08	1,00	> 0,10	> 0,10	> 0,10
**hsa-let-7c-5p**	24,99	23,82	24,73	23,61	0,66	1,00	0,70	1,00	> 0,10	> 0,10	0,0628
**hsa-let-7d-3p**	30,03	29,57	30,01	29,37	1,08	1,01	0,97	1,00	> 0,10	> 0,10	> 0,10
**hsa-let-7d-5p**	28,39	27,65	27,88	27,40	0,87	0,98	1,09	1,00	0,0846	> 0,10	> 0,10
**hsa-let-7e-3p**	35,40	34,57	34,39	33,57	0,37	0,58	0,72	1,00	> 0,10	> 0,10	> 0,10
**hsa-let-7e-5p**	27,34	26,39	26,98	26,52	0,97	1,27	1,11	1,00	> 0,10	> 0,10	> 0,10
**hsa-let-7f-1-3p**	34,74	33,89	34,69	34,14	1,13	1,38	1,04	1,00	> 0,10	> 0,10	> 0,10
**hsa-let-7f-2-3p**	32,93	33,09	33,49	33,13	1,51	1,20	1,18	1,00	> 0,10	> 0,10	> 0,10
**hsa-let-7f-5p**	26,25	25,41	25,62	25,34	0,91	1,11	1,25	1,00	> 0,10	> 0,10	> 0,10
**hsa-let-7g-3p**	33,10	33,23	33,04	34,92	5,85	4,20	3,90	1,00	> 0,10	> 0,10	> 0,10
**hsa-let-7g-5p**	24,70	23,92	24,50	23,82	0,93	1,09	0,95	1,00	> 0,10	> 0,10	> 0,10
**hsa-let-7i-3p**	35,93	35,35	35,07	35,58	1,46	1,47	1,97	1,00	> 0,10	> 0,10	> 0,10
**hsa-let-7i-5p**	28,87	28,09	28,13	27,93	0,89	1,04	1,32*	1,00	> 0,10	> 0,10	**0,0260**
**hsa-miR-100-5p**	29,75	28,58	28,19	28,83	0,91	1,39	2,37	1,00	> 0,10	> 0,10	0,0769
**hsa-miR-101-3p**	26,67	25,87	26,07	25,67	0,85	1,00	1,15	1,00	> 0,10	> 0,10	> 0,10
**hsa-miR-101-5p**	33,56	33,11	33,57	32,77	0,99	0,92	0,87	1,00	> 0,10	> 0,10	> 0,10
**hsa-miR-103a-3p**	24,53	23,77	24,28	23,52	0,85*	0,97	0,89	1,00	**0,0394**	> 0,10	> 0,10
**hsa-miR-106a-3p**	35,05	35,37	35,13	34,90	1,19	0,84	1,09	1,00	> 0,10	> 0,10	> 0,10
**hsa-miR-106a-5p**	24,17	23,51	24,02	23,13	0,83	0,89	0,81	1,00	> 0,10	> 0,10	> 0,10
**hsa-miR-106b-3p**	32,93	32,38	32,85	32,05	0,93	0,92	0,87	1,00	> 0,10	> 0,10	> 0,10
**hsa-miR-106b-5p**	27,33	26,80	27,29	26,56	1,00	0,98	0,91	1,00	> 0,10	> 0,10	> 0,10
**hsa-miR-107**	26,59	26,11	26,20	25,44	0,77	0,73	0,89	1,00	> 0,10	> 0,10	> 0,10
**hsa-miR-10a-3p**	**40,00**	36,18	35,28	35,77	0,08	0,73	1,78	1,00	> 0,10	> 0,10	> 0,10
**hsa-miR-10a-5p**	32,54	30,17	28,99	29,53	0,21*	0,74	2,21	1,00	**0,0088**	> 0,10	0,0858

hsa-miR-10b-3p	37,88	35,49	34,54	35,00	0,23	0,82	2,09	1,00	0,0695	> 0,10	> 0,10
hsa-miR-10b-5p	28,00	26,02	25,65	26,06	0,45	1,19	2,01*	1,00	> 0,10	> 0,10	**0,0266**
hsa-miR-122-5p	36,42	33,08	33,79	34,63	0,38	3,40	2,72	1,00	> 0,10	0,0513	> 0,10
hsa-miR-125a-3p	34,36	32,96	34,08	32,46	0,46*	0,82	0,49*	1,00	**0,0171**	> 0,10	**0,0327**
hsa-miR-125a-5p	25,68	24,88	25,44	24,69	0,86	1,02	0,90	1,00	> 0,10	> 0,10	> 0,10
hsa-miR-125b-2-3p	31,81	30,50	31,28	30,17	0,55	0,93	0,70**	1,00	0,0804	> 0,10	**0,0009**
hsa-miR-125b-5p	24,60	23,32	24,08	23,16	0,63	1,04	0,80	1,00	> 0,10	> 0,10	> 0,10
hsa-miR-1260a	22,63	22,46	23,11	21,75	0,93	0,71	0,59	1,00	> 0,10	> 0,10	0,0673
hsa-miR-126-3p	30,07	29,63	29,82	29,48	1,13	1,04	1,19	1,00	> 0,10	> 0,10	> 0,10
hsa-miR-126-5p	35,08	35,29	34,31	34,31	1,00	0,59	1,28	1,00	> 0,10	> 0,10	> 0,10
hsa-miR-1270	37,01	35,90	36,13	36,14	1,74	1,49	1,67	1,00	> 0,10	> 0,10	> 0,10
hsa-miR-1271-5p	36,74	35,91	36,72	36,13	0,86	1,35	1,00	1,00	> 0,10	> 0,10	> 0,10
hsa-miR-127-3p	33,55	32,17	31,80	32,64	0,92	1,62	2,73	1,00	> 0,10	> 0,10	> 0,10
hsa-miR-128-3p	31,59	30,54	31,08	31,16	1,27	1,78*	1,59*	1,00	> 0,10	**0,0195**	**0,0370**
hsa-miR-129-5p	**40,00**	36,53	36,32	36,67	0,21	2,11	2,80	1,00	> 0,10	> 0,10	> 0,10
hsa-miR-1296-5p	32,93	32,09	32,79	32,01	0,91	1,10	0,88	1,00	> 0,10	> 0,10	> 0,10
hsa-miR-130a-3p	32,89	32,12	31,94	32,51	1,32	1,52	2,26**	1,00	> 0,10	> 0,10	**0,0015**
hsa-miR-130b-3p	33,33	32,87	32,83	32,51	0,96	0,90	1,02	1,00	> 0,10	> 0,10	> 0,10
hsa-miR-130b-5p	34,39	34,75	34,57	34,57	1,94*	1,02	1,51	1,00	**0,0298**	> 0,10	> 0,10
hsa-miR-132-3p	29,72	28,58	28,97	28,55	0,76	1,14	1,13	1,00	> 0,10	> 0,10	> 0,10
hsa-miR-132-5p	35,22	34,22	34,90	33,65	0,58	0,78	0,64	1,00	> 0,10	> 0,10	> 0,10
hsa-miR-134-5p	35,34	33,43	34,44	35,30	1,28	4,25	2,74	1,00	> 0,10	0,0731	> 0,10
hsa-miR-135a-5p	26,18	25,34	25,82	25,09	0,81	0,98	0,91	1,00	> 0,10	> 0,10	> 0,10
hsa-miR-135b-5p	33,67	30,16	29,26	30,18	0,15**	1,18	2,87*	1,00	**0,0032**	> 0,10	**0,0219**
hsa-miR-136-3p	36,23	36,30	34,90	36,02	1,61	1,04	3,60	1,00	> 0,10	> 0,10	> 0,10
hsa-miR-136-5p	34,29	33,77	32,28	33,83	1,25	1,21	4,43	1,00	> 0,10	> 0,10	> 0,10
hsa-miR-138-5p	35,50	36,80	35,03	36,50	3,42	0,94	3,52	1,00	> 0,10	> 0,10	> 0,10
hsa-miR-139-3p	**40,00**	35,48	35,76	35,01	0,05	0,61	1,07	1,00	> 0,10	> 0,10	> 0,10
hsa-miR-139-5p	36,68	**40,00**	35,61	36,20	2,03	0,08	2,61	1,00	> 0,10	> 0,10	> 0,10
hsa-miR-140-3p	28,95	28,44	28,68	27,94	0,85	0,82	0,91	1,00	> 0,10	0,0958	> 0,10
hsa-miR-140-5p	31,32	30,82	31,23	30,56	1,01	0,97	0,95	1,00	> 0,10	> 0,10	> 0,10
hsa-miR-141-3p	23,91	23,30	23,44	23,00	0,92	0,94	1,12	1,00	> 0,10	> 0,10	> 0,10
hsa-miR-141-5p	30,78	30,65	30,86	30,10	1,07	0,79	0,89	1,00	> 0,10	> 0,10	0,0763
hsa-miR-142-3p	32,12	32,90	31,96	35,07	13,27*	5,21	13,05*	1,00	**0,0467**	0,0771	**0,0286**
hsa-miR-142-5p	35,19	35,85	35,86	**40,00**	54,63*	17,68**	26,67**	1,00	**0,0335**	**0,0004**	**0,0038**
hsa-miR-143-3p	35,46	35,16	35,10	36,41	4,62	2,91	4,60	1,00	> 0,10	> 0,10	> 0,10
hsa-miR-145-5p	36,89	35,09	35,62	**40,00**	14,81*	30,00	26,56	1,00	**0,0394**	0,0892	> 0,10

hsa-miR-1468-5p	34,91	34,48	34,11	33,67	0,72	0,66	0,94	1,00	> 0,10	> 0,10	> 0,10
hsa-miR-146a-5p	30,64	30,35	29,60	30,87	2,00	1,66	3,64	1,00	0,0517	> 0,10	0,0980
hsa-miR-146b-3p	**40,00**	34,42	34,85	35,50	0,08	2,15	2,41	1,00	> 0,10	> 0,10	> 0,10
hsa-miR-146b-5p	33,50	31,11	30,91	31,13	0,33*	1,18	1,77	1,00	**0,0251**	> 0,10	> 0,10
hsa-miR-148a-3p	23,61	23,98	24,22	23,59	1,30	0,88	0,98	1,00	> 0,10	> 0,10	> 0,10
hsa-miR-148b-3p	28,02	27,41	27,77	27,32	1,06	1,09	1,11	1,00	> 0,10	> 0,10	> 0,10
hsa-miR-148b-5p	34,94	34,09	34,45	33,82	0,79	0,96	0,98	1,00	> 0,10	> 0,10	> 0,10
hsa-miR-149-3p	32,41	32,41	33,17	31,95	1,24	0,84	0,65	1,00	> 0,10	> 0,10	> 0,10
hsa-miR-149-5p	26,71	25,89	26,80	25,37	0,68	0,81	0,56**	1,00	> 0,10	> 0,10	**0,0031**
hsa-miR-150-5p	34,69	34,16	**40,00**	34,46	1,01	1,29	0,03*	1,00	> 0,10	> 0,10	**0,0200**
hsa-miR-151a-3p	29,08	28,42	28,67	27,83	0,72*	0,77*	0,84*	1,00	**0,0299**	**0,0249**	**0,0368**
hsa-miR-151a-5p	26,63	25,74	26,43	25,68	0,88	1,11	0,90	1,00	> 0,10	> 0,10	> 0,10
hsa-miR-152-3p	28,76	28,38	28,39	27,90	0,94	0,83	1,08	1,00	> 0,10	> 0,10	> 0,10
hsa-miR-153-3p	32,37	31,71	32,04	31,71	1,09	1,16	1,20	1,00	> 0,10	> 0,10	> 0,10
hsa-miR-1537-3p	**40,00**	36,16	35,79	35,08	0,06*	0,56	0,79	1,00	**0,0148**	> 0,10	> 0,10
hsa-miR-154-5p	36,02	33,83	32,60	34,54	0,58	2,33	6,00	1,00	> 0,10	> 0,10	> 0,10
hsa-miR-155-5p	28,91	28,95	27,97	27,19	0,52	0,34	0,89	1,00	> 0,10	> 0,10	> 0,10
hsa-miR-15a-5p	27,14	26,11	26,34	26,19	0,88	1,22	1,36*	1,00	> 0,10	> 0,10	**0,0291**
hsa-miR-15b-3p	34,43	34,96	33,75	34,71	2,08	0,97	2,95	1,00	> 0,10	> 0,10	0,0720
hsa-miR-15b-5p	30,40	29,36	29,44	28,67	0,52	0,72	0,89	1,00	> 0,10	> 0,10	> 0,10
hsa-miR-16-1-3p	35,30	34,15	36,04	34,29	0,85	1,10	0,45	1,00	> 0,10	> 0,10	> 0,10
hsa-miR-16-2-3p	35,29	33,78	34,51	34,71	1,14	2,21	1,75	1,00	> 0,10	> 0,10	> 0,10
hsa-miR-16-5p	24,50	23,37	23,81	23,36	0,78	1,15	1,11	1,00	> 0,10	> 0,10	> 0,10
hsa-miR-17-3p	31,73	30,82	31,73	30,82	0,91	1,15	0,81	1,00	> 0,10	> 0,10	> 0,10
hsa-miR-17-5p	31,30	30,46	31,03	29,80	0,61	0,74	0,65	1,00	> 0,10	> 0,10	> 0,10
hsa-miR-181a-2-3p	36,06	34,15	34,23	33,92	0,44	0,99	1,22	1,00	> 0,10	> 0,10	> 0,10
hsa-miR-181a-5p	31,45	29,52	28,98	29,30	0,38*	1,00	1,89	1,00	**0,0188**	> 0,10	0,0878
hsa-miR-181b-5p	35,54	32,72	31,90	32,81	0,30*	1,23	2,84*	1,00	**0,0074**	> 0,10	**0,0111**
hsa-miR-181c-3p	33,17	34,51	34,45	32,80	1,02	0,35	0,48	1,00	> 0,10	> 0,10	0,0583
hsa-miR-181c-5p	34,30	34,52	33,97	34,40	1,40	1,06	1,72	1,00	> 0,10	> 0,10	> 0,10
hsa-miR-181d-5p	35,04	34,96	35,06	34,56	0,94	0,87	1,07	1,00	> 0,10	> 0,10	> 0,10
hsa-miR-182-3p	34,40	33,35	34,49	35,17	2,93*	4,10*	2,43	1,00	**0,0317**	**0,0174**	0,0514
hsa-miR-182-5p	28,04	27,13	27,80	27,28	1,01	1,28	1,06	1,00	> 0,10	> 0,10	> 0,10
hsa-miR-183-3p	35,38	34,67	35,09	34,95	0,98	1,41	1,37	1,00	> 0,10	> 0,10	> 0,10
hsa-miR-183-5p	29,62	28,54	28,84	28,73	0,93	1,33	1,40	1,00	> 0,10	> 0,10	> 0,10
hsa-miR-185-5p	27,95	27,30	27,57	26,98	0,88	0,93	1,01	1,00	> 0,10	> 0,10	> 0,10
hsa-miR-186-5p	31,96	31,44	31,22	30,85	0,79	0,77	1,17	1,00	> 0,10	> 0,10	> 0,10

**hsa-miR-187-3p**	30,69	29,01	28,67	29,47	0,74	1,59	2,65*	1,00	> 0,10	> 0,10	**0,0252**
hsa-miR-187-5p	35,06	35,81	33,73	34,85	1,69	0,60	2,79*	1,00	> 0,10	> 0,10	**0,0227**
**hsa-miR-188-3p**	34,94	35,18	35,22	34,68	1,43	0,82	1,05	1,00	> 0,10	> 0,10	> 0,10
**hsa-miR-188-5p**	36,69	34,32	34,98	34,42	0,25	1,12	0,93	1,00	> 0,10	> 0,10	> 0,10
**hsa-miR-18a-3p**	35,03	34,28	34,92	34,30	1,03	1,18	0,99	1,00	> 0,10	> 0,10	> 0,10
**hsa-miR-18a-5p**	30,87	29,89	30,56	29,91	0,88	1,17	0,96	1,00	> 0,10	> 0,10	> 0,10
**hsa-miR-18b-3p**	34,99	34,31	34,72	34,45	1,18	1,28	1,26	1,00	> 0,10	> 0,10	> 0,10
**hsa-miR-18b-5p**	30,08	29,44	29,91	29,13	0,89	0,94	0,88*	1,00	> 0,10	> 0,10	**0,0431**
**hsa-miR-1908-5p**	36,61	34,84	34,90	34,69	0,45	1,04	1,31	1,00	0,0848	> 0,10	> 0,10
**hsa-miR-190a-5p**	33,90	32,72	32,81	33,29	1,12	1,71	2,10	1,00	> 0,10	> 0,10	0,0607
**hsa-miR-190b**	**40,00**	36,65	36,64	36,63	0,20	1,90	2,17	1,00	> 0,10	> 0,10	> 0,10
**hsa-miR-1913**	33,03	33,25	33,09	33,25	1,80	0,90	1,29	1,00	> 0,10	> 0,10	> 0,10
**hsa-miR-191-3p**	35,59	34,51	36,39	34,44	0,77	1,11	0,39	1,00	> 0,10	> 0,10	> 0,10
**hsa-miR-191-5p**	26,39	25,75	26,37	25,40	0,86	0,91	0,77*	1,00	> 0,10	> 0,10	**0,0284**
**hsa-miR-192-3p**	36,65	35,95	36,55	35,16	0,47	0,58	0,58	1,00	0,0683	> 0,10	> 0,10
**hsa-miR-192-5p**	30,42	29,10	29,53	29,05	0,66	1,12	1,08	1,00	> 0,10	> 0,10	> 0,10
**hsa-miR-193a-3p**	35,68	34,60	35,12	34,73	1,01	1,27	1,16	1,00	> 0,10	> 0,10	> 0,10
**hsa-miR-193a-5p**	30,88	30,38	30,66	29,73	0,77	0,74*	0,80	1,00	> 0,10	**0,0348**	> 0,10
**hsa-miR-193b-3p**	27,59	27,15	27,59	26,72	0,93	0,86	0,83	1,00	> 0,10	> 0,10	> 0,10
**hsa-miR-193b-5p**	32,80	33,22	33,54	31,96	0,96	0,49	0,51	1,00	> 0,10	> 0,10	> 0,10
**hsa-miR-194-5p**	30,35	29,22	29,71	29,26	0,81	1,19	1,11	1,00	> 0,10	> 0,10	> 0,10
**hsa-miR-195-3p**	35,31	35,63	35,56	35,36	1,36	0,96	1,31	1,00	> 0,10	> 0,10	> 0,10
**hsa-miR-195-5p**	27,82	27,04	27,19	26,79	0,84	0,98	1,15	1,00	> 0,10	> 0,10	> 0,10
**hsa-miR-196a-5p**	37,34	35,22	36,69	35,76	0,58	1,69	0,80	1,00	> 0,10	> 0,10	> 0,10
**hsa-miR-196b-3p**	31,76	31,52	32,45	31,44	1,38	1,10	0,75	1,00	> 0,10	> 0,10	> 0,10
**hsa-miR-196b-5p**	27,54	26,90	27,67	26,66	0,93	0,98	0,76	1,00	> 0,10	> 0,10	0,0689
**hsa-miR-1972**	31,24	30,17	30,19	30,68	1,16	1,65	2,14	1,00	> 0,10	> 0,10	> 0,10
**hsa-miR-197-3p**	29,61	28,93	29,58	28,65	0,88	0,95	0,79	1,00	> 0,10	> 0,10	> 0,10
**hsa-miR-199a-3p**	35,86	35,25	35,18	35,03	0,75	1,01	1,17	1,00	> 0,10	> 0,10	> 0,10
**hsa-miR-199b-5p**	**40,00**	36,13	**40,00**	36,76	0,22	2,20	0,20	1,00	> 0,10	> 0,10	> 0,10
**hsa-miR-19a-3p**	27,60	26,91	27,25	26,46	0,78	0,85	0,88	1,00	> 0,10	> 0,10	> 0,10
**hsa-miR-19b-1-5p**	33,93	33,95	34,21	34,00	1,24	1,08	1,18	1,00	> 0,10	> 0,10	> 0,10
**hsa-miR-19b-2-5p**	36,50	37,25	36,46	35,42	0,88	0,36	0,80	1,00	> 0,10	> 0,10	> 0,10
**hsa-miR-19b-3p**	24,88	24,35	24,80	23,93	0,89	0,87	0,83	1,00	> 0,10	> 0,10	0,0844
**hsa-miR-200a-3p**	25,44	24,58	24,71	24,30	0,78	0,95	1,14	1,00	> 0,10	> 0,10	> 0,10
**hsa-miR-200a-5p**	31,73	31,11	31,21	30,71	0,84	0,88	1,07	1,00	> 0,10	> 0,10	> 0,10
**hsa-miR-200b-3p**	24,33	23,44	23,85	23,06	0,71*	0,89	0,88	1,00	**0,0088**	> 0,10	> 0,10

**hsa-miR-200b-5p**	29,29	28,47	28,93	27,95	0,68*	0,81	0,77	1,00	**0,0362**	> 0,10	> 0,10
**hsa-miR-200c-3p**	22,40	21,69	22,14	21,35	0,83	0,92	0,88	1,00	> 0,10	> 0,10	> 0,10
**hsa-miR-200c-5p**	33,22	32,57	33,62	32,93	1,07	1,74	0,94	1,00	> 0,10	0,0852	> 0,10
**hsa-miR-202-3p**	**40,00**	31,58	33,19	32,55	0,01**	2,27*	0,97	1,00	**0,0011**	**0,0449**	> 0,10
**hsa-miR-202-5p**	36,25	32,85	34,91	33,41	0,18	1,71	0,53	1,00	> 0,10	> 0,10	> 0,10
**hsa-miR-203a**	27,23	26,32	26,73	26,18	0,83	1,05	1,04	1,00	> 0,10	> 0,10	> 0,10
**hsa-miR-204-5p**	33,01	31,15	30,95	31,15	0,47*	1,16	1,74	1,00	**0,0362**	> 0,10	> 0,10
**hsa-miR-205-3p**	**40,00**	**40,00**	35,50	34,72	0,05	0,04	1,09	1,00	> 0,10	> 0,10	> 0,10
**hsa-miR-205-5p**	28,17	26,15	25,09	26,08	0,40	1,11	3,00*	1,00	0,0569	> 0,10	**0,0164**
**hsa-miR-20a-3p**	31,00	30,27	31,05	30,00	0,86	0,96	0,73	1,00	> 0,10	> 0,10	> 0,10
**hsa-miR-20a-5p**	25,01	24,26	24,98	23,92	0,81*	0,91	0,73*	1,00	**0,0196**	> 0,10	**0,0161**
**hsa-miR-20b-3p**	31,85	31,21	31,73	30,85	0,85	0,90	0,82	1,00	> 0,10	> 0,10	> 0,10
**hsa-miR-20b-5p**	29,55	29,30	29,42	28,31	0,73	0,58	0,70	1,00	> 0,10	> 0,10	> 0,10
**hsa-miR-210-3p**	29,91	28,91	29,47	28,89	0,85	1,15	1,02	1,00	> 0,10	> 0,10	> 0,10
**hsa-miR-2110**	31,81	31,42	32,26	31,42	1,31	1,16	0,84	1,00	> 0,10	> 0,10	> 0,10
**hsa-miR-212-3p**	34,74	33,62	34,63	33,43	0,53	1,02	0,66	1,00	> 0,10	> 0,10	> 0,10
hsa-miR-212-5p	36,52	36,35	**40,00**	**40,00**	14,68**	12,50**	1,51	1,00	**0,0082**	**0,0006**	> 0,10
**hsa-miR-21-3p**	33,78	33,94	33,63	33,20	1,15	0,69	1,12	1,00	> 0,10	> 0,10	> 0,10
**hsa-miR-215-5p**	31,72	30,50	30,69	30,62	0,80	1,26	1,44	1,00	> 0,10	> 0,10	> 0,10
**hsa-miR-21-5p**	22,72	22,42	22,74	22,00	1,04	0,87	0,91	1,00	> 0,10	> 0,10	> 0,10
**hsa-miR-216a-5p**	35,88	35,21	34,55	35,61	1,63	1,14	3,72*	1,00	> 0,10	> 0,10	**0,0344**
hsa-miR-217	**40,00**	**40,00**	36,57	35,66	0,09*	0,06*	0,82	1,00	**0,0137**	**0,0209**	> 0,10
**hsa-miR-218-5p**	34,02	33,55	32,69	33,50	1,19	1,12	2,65	1,00	> 0,10	> 0,10	> 0,10
**hsa-miR-219a-5p**	33,83	33,67	34,01	33,34	1,22	0,92	0,96	1,00	> 0,10	> 0,10	> 0,10
**hsa-miR-221-3p**	31,92	29,00	28,11	28,80	0,20**	1,01	2,44	1,00	**0,0048**	> 0,10	> 0,10
hsa-miR-222-3p	30,00	27,13	26,03	27,32	0,27*	1,32	3,70*	1,00	**0,0398**	0,0998	**0,0092**
hsa-miR-223-3p	29,99	30,11	29,32	33,05	14,32*	8,93	20,12*	1,00	**0,0363**	0,0525	**0,0158**
**hsa-miR-22-3p**	28,04	27,67	27,50	27,41	1,10	0,97	1,42	1,00	> 0,10	> 0,10	> 0,10
**hsa-miR-224-3p**	34,28	34,05	33,41	33,95	0,88	0,78	1,57	1,00	> 0,10	> 0,10	> 0,10
**hsa-miR-224-5p**	36,17	34,81	34,92	35,71	1,24	1,86	2,62	1,00	> 0,10	> 0,10	0,0659
**hsa-miR-22-5p**	30,01	29,62	29,51	29,45	1,16	1,03	1,45	1,00	> 0,10	> 0,10	> 0,10
**hsa-miR-23a-3p**	25,86	25,34	25,73	24,95	0,91	0,88	0,88	1,00	> 0,10	> 0,10	> 0,10
**hsa-miR-23b-3p**	25,55	25,23	25,46	24,37	0,76	0,64	0,71	1,00	> 0,10	0,0537	> 0,10
**hsa-miR-23b-5p**	35,35	34,53	35,65	36,04	2,76	2,44	1,67	1,00	> 0,10	> 0,10	> 0,10
**hsa-miR-24-1-5p**	34,78	35,66	35,12	34,47	1,06	0,51	0,96	1,00	> 0,10	> 0,10	> 0,10
**hsa-miR-24-3p**	25,61	25,29	25,41	24,57	0,83	0,70	0,84	1,00	> 0,10	> 0,10	> 0,10
**hsa-miR-25-3p**	27,57	26,92	27,63	26,76	0,98	1,04	0,83	1,00	> 0,10	> 0,10	> 0,10

**hsa-miR-25-5p**	36,03	35,83	37,61	35,71	1,37	1,07	0,41	1,00	> 0,10	> 0,10	> 0,10
**hsa-miR-26a-1-3p**	36,48	35,01	36,58	35,41	0,81	1,53	0,67	1,00	> 0,10	> 0,10	> 0,10
**hsa-miR-26a-5p**	24,99	24,37	24,70	23,68	0,69	0,72	0,75	1,00	> 0,10	0,0693	0,0710
**hsa-miR-26b-3p**	33,82	32,99	33,98	32,88	0,89	1,07	0,70	1,00	> 0,10	> 0,10	> 0,10
**hsa-miR-26b-5p**	26,28	25,84	25,98	24,90	0,66	0,60	0,72	1,00	> 0,10	> 0,10	> 0,10
**hsa-miR-27a-3p**	29,52	29,41	29,27	28,53	0,86	0,63	0,90	1,00	> 0,10	> 0,10	> 0,10
**hsa-miR-27b-3p**	26,67	26,40	26,56	25,64	0,84	0,68	0,80	1,00	> 0,10	0,0672	> 0,10
**hsa-miR-27b-5p**	35,56	35,87	35,57	34,80	1,01	0,55	0,75	1,00	> 0,10	> 0,10	> 0,10
**hsa-miR-28-3p**	28,28	27,60	28,57	27,33	0,88	0,96	0,64	1,00	> 0,10	> 0,10	0,0567
**hsa-miR-28-5p**	28,05	27,24	28,11	26,95	0,80	0,95	0,68	1,00	> 0,10	> 0,10	0,0938
**hsa-miR-296-5p**	35,09	34,89	34,84	34,72	1,02	1,20	1,17	1,00	> 0,10	> 0,10	> 0,10
**hsa-miR-29a-3p**	27,01	26,66	26,99	26,37	1,10	0,94	0,98	1,00	> 0,10	> 0,10	> 0,10
**hsa-miR-29a-5p**	30,21	29,96	30,30	29,09	0,79	0,64	0,66	1,00	0,0538	0,0539	0,0563
**hsa-miR-29b-2-5p**	30,79	30,14	30,37	29,91	0,93	0,99	1,10	1,00	> 0,10	> 0,10	> 0,10
**hsa-miR-29b-3p**	26,57	26,02	26,21	25,67	0,92	0,91	1,04	1,00	> 0,10	> 0,10	> 0,10
**hsa-miR-29c-3p**	25,77	25,32	25,67	25,18	1,13	1,05	1,07	1,00	> 0,10	> 0,10	> 0,10
**hsa-miR-29c-5p**	31,84	31,30	31,66	31,30	1,18	1,15	1,18	1,00	> 0,10	> 0,10	> 0,10
**hsa-miR-301a-3p**	33,03	31,30	31,97	31,33	0,53	1,19	0,97	1,00	> 0,10	> 0,10	> 0,10
**hsa-miR-301b**	35,64	35,11	35,09	35,14	0,93	1,18	1,57	1,00	> 0,10	> 0,10	> 0,10
**hsa-miR-30a-3p**	29,79	29,11	29,60	28,61	0,76	0,82	0,76	1,00	> 0,10	> 0,10	> 0,10
**hsa-miR-30a-5p**	27,96	27,59	27,87	26,92	0,84	0,73	0,79	1,00	> 0,10	> 0,10	> 0,10
**hsa-miR-30b-3p**	35,75	35,60	36,07	35,79	1,21	1,19	1,13	1,00	> 0,10	> 0,10	> 0,10
**hsa-miR-30b-5p**	24,94	24,44	25,07	24,05	0,93	0,88	0,75*	1,00	> 0,10	> 0,10	**0,0225**
**hsa-miR-30c-1-3p**	35,72	36,63	36,57	35,92	1,51	0,71	0,70	1,00	> 0,10	> 0,10	> 0,10
**hsa-miR-30c-2-3p**	33,85	33,37	34,48	33,53	0,95	1,01	0,60	1,00	> 0,10	> 0,10	> 0,10
**hsa-miR-30c-5p**	25,88	25,22	25,86	25,02	0,94	1,01	0,85	1,00	> 0,10	> 0,10	> 0,10
**hsa-miR-30d-3p**	31,90	31,45	32,05	30,91	0,86	0,80	0,69	1,00	> 0,10	> 0,10	0,0992
**hsa-miR-30d-5p**	26,12	25,42	25,81	25,04	0,81	0,89	0,89	1,00	> 0,10	> 0,10	> 0,10
**hsa-miR-30e-3p**	28,39	27,86	28,32	27,65	1,03	1,01	0,96	1,00	> 0,10	> 0,10	> 0,10
**hsa-miR-30e-5p**	26,84	26,34	26,68	25,89	0,88	0,85	0,88	1,00	> 0,10	> 0,10	> 0,10
**hsa-miR-31-3p**	35,45	33,42	32,79	33,59	0,36	1,31	2,65	1,00	> 0,10	> 0,10	0,0857
**hsa-miR-31-5p**	31,73	29,35	28,48	29,53	0,37*	1,31	3,14*	1,00	**0,0470**	> 0,10	**0,0220**
**hsa-miR-320a**	26,52	25,81	26,17	25,59	0,90	0,99	1,01	1,00	> 0,10	> 0,10	> 0,10
**hsa-miR-320b**	27,95	27,32	27,83	27,11	0,95	1,00	0,92	1,00	> 0,10	> 0,10	> 0,10
**hsa-miR-320c**	29,70	29,12	29,57	28,77	0,90	0,91	0,87	1,00	> 0,10	> 0,10	> 0,10
**hsa-miR-320d**	30,93	29,85	30,66	29,94	1,46	1,23	0,91	1,00	> 0,10	> 0,10	> 0,10
**hsa-miR-323a-3p**	36,51	34,74	34,62	35,21	0,80	1,61	1,92	1,00	> 0,10	> 0,10	> 0,10

hsa-miR-32-3p	35,15	33,97	35,17	34,43	1,04	1,60	0,91	1,00	> 0,10	> 0,10	> 0,10
hsa-miR-324-3p	29,12	28,51	28,94	28,41	1,05	1,08	1,05	1,00	> 0,10	> 0,10	> 0,10
hsa-miR-324-5p	29,44	28,49	29,06	28,36	0,81	1,06	0,93	1,00	> 0,10	> 0,10	> 0,10
hsa-miR-32-5p	29,00	28,24	28,53	28,40	1,13	1,30	1,38	1,00	> 0,10	> 0,10	0,0747
hsa-miR-326	34,44	33,53	34,19	33,04	0,42	0,69	0,81	1,00	> 0,10	> 0,10	> 0,10
hsa-miR-328-3p	31,51	30,89	31,38	30,80	1,04	1,09	1,01	1,00	> 0,10	> 0,10	> 0,10
hsa-miR-330-3p	35,07	34,00	33,48	33,37	0,53	0,75	1,41	1,00	0,0845	> 0,10	> 0,10
hsa-miR-331-3p	30,11	29,16	29,59	29,14	0,87	1,14	1,11	1,00	> 0,10	> 0,10	> 0,10
hsa-miR-331-5p	35,68	35,23	35,96	35,33	1,00	1,04	0,95	1,00	> 0,10	> 0,10	> 0,10
hsa-miR-335-3p	36,54	35,56	35,97	35,98	1,17	1,56	1,52	1,00	> 0,10	> 0,10	> 0,10
hsa-miR-335-5p	31,80	31,39	31,98	31,10	1,05	0,94	0,82	1,00	> 0,10	> 0,10	> 0,10
hsa-miR-338-3p	30,50	29,36	30,29	29,57	0,90	1,35	0,92	1,00	> 0,10	> 0,10	> 0,10
hsa-miR-339-3p	31,09	30,55	31,15	30,37	1,04	1,02	0,88	1,00	> 0,10	> 0,10	> 0,10
hsa-miR-339-5p	29,84	29,49	29,84	29,52	1,38	1,19	1,21	1,00	> 0,10	> 0,10	> 0,10
hsa-miR-33a-3p	32,94	32,00	32,77	31,70	0,72	0,94	0,72	1,00	> 0,10	> 0,10	0,0648
hsa-miR-33a-5p	29,72	29,34	29,37	28,85	0,94	0,83	1,06	1,00	> 0,10	> 0,10	> 0,10
hsa-miR-33b-3p	33,92	33,93	34,25	33,34	0,88	0,89	0,81	1,00	> 0,10	> 0,10	> 0,10
hsa-miR-33b-5p	34,81	34,47	34,55	35,73	2,92	2,50	3,10	1,00	> 0,10	> 0,10	> 0,10
hsa-miR-340-3p	33,87	33,15	34,32	33,11	0,78	1,13	0,66	1,00	> 0,10	> 0,10	> 0,10
hsa-miR-340-5p	31,92	31,60	32,22	31,38	0,92	1,01	0,86	1,00	> 0,10	> 0,10	> 0,10
hsa-miR-342-3p	27,45	26,69	27,28	25,96	0,61*	0,70*	0,61**	1,00	**0,0376**	**0,0389**	**0,0032**
hsa-miR-342-5p	35,17	34,06	34,23	33,80	0,66	0,97	1,12	1,00	> 0,10	> 0,10	> 0,10
hsa-miR-345-5p	29,28	28,42	28,30	28,35	0,90	1,10	1,57*	1,00	> 0,10	> 0,10	**0,0273**
hsa-miR-34a-5p	27,95	27,76	28,00	28,50	2,50	1,93	2,14	1,00	> 0,10	0,0980	0,0717
hsa-miR-34b-3p	**40,00**	33,34	33,64	33,35	0,02**	1,17	1,24	1,00	**0,0010**	> 0,10	> 0,10
hsa-miR-34b-5p	**40,00**	30,29	30,42	31,64	0,01**	2,95	3,51	1,00	**0,0004**	0,0670	0,0965
hsa-miR-34c-3p	36,61	34,47	33,75	34,57	0,32	1,25	2,67	1,00	> 0,10	> 0,10	> 0,10
hsa-miR-34c-5p	37,16	30,59	30,07	31,21	0,03**	1,77	3,32	1,00	**0,0035**	> 0,10	> 0,10
hsa-miR-361-3p	31,06	30,77	30,85	30,30	1,01	0,84	1,04	1,00	> 0,10	> 0,10	> 0,10
hsa-miR-361-5p	26,85	26,46	26,92	25,97	0,93	0,83	0,78	1,00	> 0,10	> 0,10	> 0,10
hsa-miR-362-3p	32,18	31,08	31,51	30,93	0,72	1,04	1,01	1,00	> 0,10	> 0,10	> 0,10
hsa-miR-362-5p	34,56	33,14	33,37	33,32	0,72	1,31	1,46	1,00	> 0,10	> 0,10	> 0,10
hsa-miR-363-3p	25,84	25,51	25,70	24,90	0,89	0,76	0,87	1,00	> 0,10	> 0,10	> 0,10
hsa-miR-363-5p	34,34	35,00	33,96	34,70	2,19	0,94	2,12	1,00	> 0,10	> 0,10	> 0,10
hsa-miR-365a-3p	29,38	28,81	29,71	28,78	1,13	1,13	0,79**	1,00	> 0,10	> 0,10	**0,0020**
hsa-miR-365b-5p	36,86	35,98	36,12	35,64	0,57	0,93	1,10	1,00	> 0,10	> 0,10	> 0,10
hsa-miR-369-3p	36,32	**40,00**	35,85	35,83	0,95	0,07**	1,28	1,00	> 0,10	**0,0032**	> 0,10

hsa-miR-370-3p	35,47	34,55	34,81	**40,00**	30,24	50,73**	46,53	1,00	0,0582	**0,0006**	> 0,10
**hsa-miR-374a-5p**	29,76	29,30	29,85	28,62	0,78	0,72	0,64	1,00	> 0,10	> 0,10	0,0881
**hsa-miR-374b-3p**	34,32	33,98	34,37	33,27	0,83	0,71	0,71	1,00	> 0,10	> 0,10	> 0,10
hsa-miR-374b-5p	28,59	28,19	28,83	27,49	0,80*	0,71	0,60**	1,00	**0,0449**	0,0773	**0,0010**
**hsa-miR-375**	22,74	22,19	22,77	21,55	0,75	0,74	0,65	1,00	> 0,10	> 0,10	> 0,10
**hsa-miR-376a-3p**	34,74	33,30	32,91	33,89	0,95	1,74	3,00	1,00	> 0,10	> 0,10	> 0,10
**hsa-miR-376a-5p**	**40,00**	36,10	36,00	36,19	0,13*	1,34	1,58	1,00	**0,0132**	> 0,10	> 0,10
**hsa-miR-376b-3p**	36,95	35,28	34,55	36,59	1,45	3,15	5,74	1,00	> 0,10	> 0,10	> 0,10
**hsa-miR-376c-3p**	33,50	32,32	32,71	33,82	1,64	3,29*	3,29	1,00	> 0,10	**0,0116**	> 0,10
**hsa-miR-377-3p**	36,45	36,51	34,83	37,36	2,51	2,13	6,43	1,00	> 0,10	> 0,10	> 0,10
**hsa-miR-378a-5p**	36,37	36,04	37,17	36,20	1,27	1,41	0,84	1,00	> 0,10	> 0,10	> 0,10
**hsa-miR-379-5p**	35,68	33,55	34,13	34,76	1,03	2,69	1,98	1,00	> 0,10	> 0,10	> 0,10
**hsa-miR-381-3p**	36,62	36,84	35,88	36,86	1,31	0,85	2,53	1,00	> 0,10	> 0,10	> 0,10
**hsa-miR-382-3p**	**40,00**	35,96	35,87	36,79	0,17*	1,39	2,19	1,00	**0,0197**	> 0,10	> 0,10
**hsa-miR-382-5p**	34,51	34,01	34,01	34,51	1,71	1,63	2,13	1,00	> 0,10	> 0,10	> 0,10
**hsa-miR-409-3p**	34,73	33,42	33,54	34,08	1,25	1,84	2,20	1,00	> 0,10	> 0,10	> 0,10
**hsa-miR-410-3p**	36,62	35,95	33,93	36,58	2,83	1,79	6,84	1,00	> 0,10	> 0,10	> 0,10
**hsa-miR-411-5p**	35,91	33,84	34,27	35,24	0,82	3,07	2,97	1,00	> 0,10	> 0,10	> 0,10
**hsa-miR-421**	31,66	30,84	31,28	30,35	0,69	0,82	0,80	1,00	> 0,10	0,0545	> 0,10
**hsa-miR-423-3p**	27,30	26,71	27,27	26,57	1,03	1,05	0,93	1,00	> 0,10	> 0,10	> 0,10
**hsa-miR-423-5p**	27,84	27,26	27,84	27,21	1,11	1,12	0,98	1,00	> 0,10	> 0,10	> 0,10
**hsa-miR-424-3p**	**40,00**	34,45	33,39	34,38	0,03*	1,11	3,02	1,00	**0,0068**	> 0,10	> 0,10
**hsa-miR-424-5p**	34,21	31,30	30,03	31,14	0,20	1,03	3,28	1,00	0,0869	> 0,10	> 0,10
**hsa-miR-425-3p**	31,59	31,20	31,11	30,46	0,79	0,69*	0,97	1,00	> 0,10	**0,0166**	> 0,10
**hsa-miR-425-5p**	29,31	28,61	28,99	28,45	0,95	1,04	1,04	1,00	> 0,10	> 0,10	> 0,10
**hsa-miR-429**	29,04	28,13	28,23	27,56	0,61	0,78	0,95	1,00	> 0,10	> 0,10	> 0,10
**hsa-miR-432-5p**	36,68	35,53	36,15	36,17	2,08	1,84*	1,56	1,00	> 0,10	**0,0419**	> 0,10
**hsa-miR-449a**	35,34	30,66	31,17	31,45	0,12*	2,00	1,84	1,00	**0,0372**	> 0,10	> 0,10
**hsa-miR-449b-3p**	**40,00**	36,57	36,85	35,74	0,08	0,47	0,59	1,00	> 0,10	> 0,10	> 0,10
**hsa-miR-449b-5p**	**40,00**	35,47	35,89	34,77	0,06	0,65	0,86	1,00	> 0,10	> 0,10	> 0,10
**hsa-miR-450a-5p**	**40,00**	34,57	34,08	35,00	0,05*	1,57	2,86	1,00	**0,0063**	> 0,10	> 0,10
**hsa-miR-451a**	35,60	35,59	35,44	35,06	0,91	0,81	1,18	1,00	> 0,10	> 0,10	> 0,10
**hsa-miR-452-5p**	35,39	33,80	34,29	33,61	0,50*	1,01	0,94	1,00	**0,0500**	> 0,10	> 0,10
**hsa-miR-454-3p**	31,52	30,66	31,34	30,68	0,95	1,17	0,96	1,00	> 0,10	> 0,10	> 0,10
**hsa-miR-455-3p**	32,48	30,42	30,40	31,10	0,66	1,86	2,47*	1,00	> 0,10	> 0,10	**0,0080**
**hsa-miR-455-5p**	35,35	33,50	33,61	34,83	0,91	2,90*	3,52*	1,00	> 0,10	**0,0472**	**0,0478**
**hsa-miR-484**	30,33	29,35	29,95	29,34	0,86	1,15	0,99	1,00	> 0,10	> 0,10	> 0,10

**hsa-miR-485-3p**	36,34	36,14	34,09	34,34	0,47	0,32	1,76	1,00	> 0,10	> 0,10	> 0,10
**hsa-miR-486-5p**	34,96	34,64	34,40	32,53	0,32	0,27	0,41	1,00	> 0,10	> 0,10	> 0,10
**hsa-miR-487b-3p**	35,25	34,87	34,02	34,89	1,34	1,18	2,77	1,00	> 0,10	> 0,10	> 0,10
**hsa-miR-491-5p**	31,31	31,03	31,21	30,78	1,18	0,98	1,12	1,00	> 0,10	> 0,10	> 0,10
**hsa-miR-493-3p**	35,75	34,14	35,11	35,16	0,87	1,74	1,58	1,00	> 0,10	> 0,10	> 0,10
**hsa-miR-493-5p**	**40,00**	35,77	35,50	36,90	0,24	3,10	4,89	1,00	> 0,10	> 0,10	> 0,10
**hsa-miR-495-3p**	35,77	34,77	34,07	34,73	0,58	1,02	2,16	1,00	> 0,10	> 0,10	> 0,10
**hsa-miR-497-5p**	28,64	27,76	28,19	28,03	1,12	1,40	1,36	1,00	> 0,10	> 0,10	0,0900
**hsa-miR-499a-5p**	35,81	34,58	34,02	33,73	0,32	0,65	1,26	1,00	> 0,10	> 0,10	> 0,10
**hsa-miR-500a-5p**	33,82	32,23	31,02	31,60	0,37*	0,75	2,26*	1,00	**0,0460**	> 0,10	**0,0464**
**hsa-miR-501-3p**	33,80	32,20	32,32	32,14	0,54	1,12	1,34	1,00	> 0,10	> 0,10	0,0941
**hsa-miR-501-5p**	34,26	31,97	31,39	32,23	0,42	1,39	2,72	1,00	> 0,10	> 0,10	0,0562
**hsa-miR-502-3p**	32,98	31,36	30,87	31,42	0,58*	1,21	1,87	1,00	**0,0478**	> 0,10	> 0,10
**hsa-miR-503-5p**	**40,00**	35,83	**40,00**	35,27	0,07	0,69	0,06	1,00	0,0828	> 0,10	0,0951
**hsa-miR-504-5p**	34,13	33,70	34,55	33,88	1,44	1,31	0,80	1,00	> 0,10	> 0,10	> 0,10
**hsa-miR-505-3p**	32,51	31,57	31,53	31,24	0,71	0,92	1,24	1,00	> 0,10	> 0,10	> 0,10
**hsa-miR-506-3p**	**40,00**	36,67	36,07	36,51	0,15*	1,21	2,42	1,00	**0,0068**	> 0,10	> 0,10
**hsa-miR-507**	**40,00**	35,09	35,39	35,05	0,06**	1,12	1,20	1,00	**0,0025**	> 0,10	> 0,10
**hsa-miR-508-3p**	**40,00**	34,80	34,69	34,92	0,05*	1,26	2,10	1,00	**0,0079**	> 0,10	> 0,10
**hsa-miR-508-5p**	**40,00**	35,70	35,23	37,22	0,25*	2,93	7,23	1,00	**0,0233**	> 0,10	> 0,10
**hsa-miR-509-3-5p**	**40,00**	32,63	33,00	32,78	0,01**	1,28	1,30	1,00	**0,0008**	> 0,10	> 0,10
**hsa-miR-510-5p**	**40,00**	33,61	33,70	35,25	0,06*	3,61	5,23	1,00	**0,0277**	> 0,10	> 0,10
**hsa-miR-513a-3p**	**40,00**	35,91	36,06	36,25	0,14*	1,38	2,23	1,00	**0,0348**	> 0,10	> 0,10
**hsa-miR-513b-5p**	36,26	33,87	33,92	35,82	0,97	3,85*	6,66*	1,00	> 0,10	**0,0145**	**0,0108**
**hsa-miR-513c-5p**	**40,00**	32,53	32,87	33,18	0,02**	1,81	2,20	1,00	**0,0008**	> 0,10	> 0,10
**hsa-miR-514a-3p**	**40,00**	30,53	30,41	30,76	0,00**	1,23	2,05	1,00	**0,0014**	> 0,10	> 0,10
**hsa-miR-515-5p**	36,54	35,85	36,42	36,79	1,53	2,51	1,87	1,00	> 0,10	> 0,10	> 0,10
**hsa-miR-517a-3p**	**40,00**	33,45	34,20	33,71	0,02**	1,38	1,08	1,00	**0,0005**	> 0,10	> 0,10
**hsa-miR-517c-3p**	**40,00**	34,20	34,87	34,45	0,04**	1,38	1,13	1,00	**0,0013**	> 0,10	> 0,10
**hsa-miR-518e-3p**	**40,00**	34,96	36,03	36,45	0,15*	3,26	2,02	1,00	**0,0440**	> 0,10	> 0,10
**hsa-miR-518e-5p**	**40,00**	35,50	35,89	35,46	0,08*	1,23	1,44	1,00	**0,0063**	> 0,10	> 0,10
**hsa-miR-518f-5p**	35,00	35,43	35,73	35,83	6,34	2,17	1,99	1,00	> 0,10	> 0,10	> 0,10
**hsa-miR-519a-3p**	**40,00**	34,71	35,97	35,61	0,08**	2,19	1,20	1,00	**0,0044**	0,0591	> 0,10
**hsa-miR-519d-3p**	**40,00**	35,35	35,46	35,65	0,08**	1,45	1,76	1,00	**0,0047**	> 0,10	> 0,10
**hsa-miR-520g-3p**	**40,00**	35,83	36,82	**40,00**	1,71	20,87*	16,15*	1,00	> 0,10	**0,0195**	**0,0417**
**hsa-miR-520h**	**40,00**	36,05	35,50	**40,00**	1,71	20,86*	40,29**	1,00	> 0,10	**0,0184**	**0,0002**
**hsa-miR-522-3p**	**40,00**	34,50	35,26	36,13	0,13*	3,91	2,54	1,00	**0,0311**	0,0762	> 0,10

**hsa-miR-532-3p**	30,07	28,98	29,46	28,60	0,62*	0,89	0,83	1,00	**0,0421**	0,0983	> 0,10
**hsa-miR-532-5p**	30,32	29,22	29,49	28,98	0,68**	0,98	1,07	1,00	**0,0019**	> 0,10	> 0,10
**hsa-miR-539-5p**	34,97	34,29	32,98	34,83	1,08	1,52	3,55	1,00	> 0,10	> 0,10	> 0,10
**hsa-miR-542-5p**	**40,00**	34,68	33,94	34,55	0,04	1,08	2,36	1,00	0,0892	> 0,10	> 0,10
**hsa-miR-543**	35,21	35,57	34,13	35,67	1,97	1,36	4,80	1,00	> 0,10	> 0,10	0,0705
**hsa-miR-548j-5p**	37,04	36,42	36,50	34,65	0,31	0,29	0,25	1,00	> 0,10	> 0,10	> 0,10
**hsa-miR-548k**	36,60	35,45	34,54	35,89	0,88	1,72	3,55*	1,00	> 0,10	> 0,10	**0,0258**
**hsa-miR-550a-3p**	36,33	36,30	35,66	37,16	2,37	2,15*	3,15	1,00	> 0,10	**0,0100**	> 0,10
**hsa-miR-551b-3p**	**40,00**	34,61	34,56	34,55	0,04**	1,11	1,51	1,00	**0,0009**	> 0,10	> 0,10
**hsa-miR-570-3p**	35,56	33,54	35,08	35,44	1,43	3,90	1,76	1,00	> 0,10	> 0,10	> 0,10
**hsa-miR-574-3p**	26,43	25,97	26,82	25,37	0,82	0,77	0,55	1,00	> 0,10	> 0,10	0,0721
**hsa-miR-576-5p**	37,56	37,79	36,17	35,83	0,59	0,30	1,20	1,00	> 0,10	0,0508	> 0,10
**hsa-miR-582-3p**	32,64	32,79	**40,00**	32,72	1,25	1,00	0,01**	1,00	> 0,10	> 0,10	**0,0005**
**hsa-miR-582-5p**	29,27	28,68	29,33	28,67	1,13	1,16	0,96	1,00	> 0,10	> 0,10	> 0,10
**hsa-miR-584-5p**	37,07	36,46	34,99	35,15	0,29	0,39	1,42	1,00	> 0,10	> 0,10	> 0,10
**hsa-miR-589-3p**	35,40	33,54	34,51	32,92	0,31	0,75	0,51	1,00	> 0,10	> 0,10	> 0,10
**hsa-miR-589-5p**	35,50	35,06	35,57	34,42	0,62	0,74	0,68	1,00	> 0,10	> 0,10	> 0,10
**hsa-miR-590-3p**	33,22	32,44	33,50	32,22	0,85	0,99	0,62	1,00	> 0,10	> 0,10	> 0,10
**hsa-miR-590-5p**	29,62	28,90	29,45	28,61	0,85	0,95	0,85	1,00	> 0,10	> 0,10	> 0,10
**hsa-miR-592**	35,97	35,88	35,45	35,73	1,13	1,06	1,58	1,00	> 0,10	> 0,10	> 0,10
**hsa-miR-598-3p**	30,79	30,35	30,91	30,06	1,03	0,94	0,84	1,00	> 0,10	> 0,10	> 0,10
**hsa-miR-615-3p**	36,71	35,85	35,60	35,71	0,67	1,24	1,64	1,00	> 0,10	> 0,10	> 0,10
**hsa-miR-616-5p**	**40,00**	36,37	37,53	35,89	0,10	0,84	0,42	1,00	> 0,10	> 0,10	> 0,10
**hsa-miR-618**	35,93	36,88	**40,00**	35,46	0,92	0,57	0,06	1,00	> 0,10	> 0,10	> 0,10
**hsa-miR-625-3p**	36,57	35,59	35,70	35,64	0,69	1,20	1,45	1,00	> 0,10	> 0,10	> 0,10
**hsa-miR-627-5p**	35,51	34,65	35,00	34,39	0,79	0,97	0,99	1,00	> 0,10	> 0,10	> 0,10
**hsa-miR-628-3p**	33,96	33,34	33,64	33,38	1,14	1,19	1,26	1,00	> 0,10	> 0,10	> 0,10
**hsa-miR-628-5p**	35,71	35,11	35,76	35,33	1,00	1,35	1,12	1,00	> 0,10	> 0,10	> 0,10
**hsa-miR-629-3p**	36,58	34,86	35,33	34,26	0,39	0,77	0,72	1,00	> 0,10	> 0,10	> 0,10
**hsa-miR-629-5p**	31,91	31,37	32,12	31,08	0,97	0,95	0,74	1,00	> 0,10	> 0,10	> 0,10
**hsa-miR-641**	**40,00**	36,21	36,25	36,74	0,15	1,21	2,53	1,00	> 0,10	> 0,10	> 0,10
**hsa-miR-642a-5p**	**40,00**	36,42	36,46	37,51	0,31	2,48	3,17	1,00	0,0707	> 0,10	0,0878
**hsa-miR-645**	**40,00**	35,16	35,20	35,82	0,08	2,08	1,65	1,00	> 0,10	> 0,10	> 0,10
**hsa-miR-651-5p**	33,82	33,71	34,53	33,92	1,83	1,34	0,99	1,00	> 0,10	> 0,10	> 0,10
**hsa-miR-652-3p**	29,77	28,70	28,97	28,50	0,71	1,01	1,09	1,00	> 0,10	> 0,10	> 0,10
**hsa-miR-660-5p**	29,49	28,14	27,99	27,94	0,58*	1,01	1,46	1,00	**0,0097**	> 0,10	> 0,10
**hsa-miR-663a**	31,11	30,93	30,86	30,31	0,99	0,76	1,04	1,00	> 0,10	> 0,10	> 0,10

**hsa-miR-664a-3p**	32,41	31,58	32,41	31,56	0,95	1,15	0,84	1,00	> 0,10	> 0,10	> 0,10
**hsa-miR-671-3p**	33,92	33,32	34,16	33,14	1,00	1,02	0,75	1,00	> 0,10	> 0,10	> 0,10
**hsa-miR-671-5p**	36,33	33,72	34,94	34,44	0,36	1,42	0,90	1,00	> 0,10	> 0,10	> 0,10
**hsa-miR-708-3p**	36,23	36,82	36,93	36,71	4,08	1,45	1,31	1,00	> 0,10	> 0,10	> 0,10
**hsa-miR-708-5p**	34,91	35,80	35,62	35,32	1,74	0,83	1,23	1,00	> 0,10	> 0,10	> 0,10
**hsa-miR-7-1-3p**	31,41	30,27	31,24	29,84	0,58	0,86	0,57	1,00	> 0,10	> 0,10	> 0,10
**hsa-miR-744-3p**	34,69	34,24	34,86	33,55	0,78	0,72	0,61	1,00	> 0,10	> 0,10	> 0,10
**hsa-miR-744-5p**	31,47	31,04	31,47	30,62	0,95	0,86	0,84	1,00	> 0,10	> 0,10	> 0,10
**hsa-miR-7-5p**	32,78	31,71	32,37	32,08	1,05	1,49	1,23	1,00	> 0,10	> 0,10	> 0,10
**hsa-miR-766-3p**	32,07	31,25	32,11	31,02	0,83	0,99	0,71	1,00	> 0,10	> 0,10	0,0965
**hsa-miR-769-5p**	31,99	31,45	32,03	31,38	1,12	1,10	0,97	1,00	> 0,10	> 0,10	> 0,10
**hsa-miR-873-5p**	**40,00**	36,03	36,61	**40,00**	1,71	18,17*	15,86**	1,00	> 0,10	**0,0070**	**0,0017**
**hsa-miR-877-5p**	34,49	32,38	32,21	32,69	0,38	1,44	2,11*	1,00	> 0,10	0,0645	**0,0155**
**hsa-miR-885-3p**	34,79	33,95	34,22	34,24	0,87	1,18	1,08	1,00	> 0,10	> 0,10	> 0,10
**hsa-miR-887-3p**	36,49	36,22	35,58	35,53	0,68	0,71	1,05	1,00	> 0,10	> 0,10	> 0,10
**hsa-miR-888-3p**	**40,00**	35,27	35,05	35,01	0,05**	0,97	1,47	1,00	**0,0018**	> 0,10	> 0,10
**hsa-miR-888-5p**	34,43	26,73	25,96	26,79	0,01**	1,21	2,68	1,00	**0,0013**	> 0,10	0,0613
**hsa-miR-890**	35,61	27,48	26,64	27,99	0,01	1,66	3,85*	1,00	0,0828	0,0703	**0,0320**
**hsa-miR-891a-5p**	31,82	24,58	23,77	24,62	0,01*	1,20	2,74	1,00	**0,0208**	> 0,10	0,0640
**hsa-miR-891b**	35,92	29,34	28,69	29,61	0,02*	1,40	2,86	1,00	**0,0129**	> 0,10	0,0751
**hsa-miR-892a**	**40,00**	30,29	29,40	29,89	0,00**	0,88	2,13	1,00	**0,0001**	> 0,10	> 0,10
**hsa-miR-92a-1-5p**	35,02	34,84	36,56	34,76	1,43	0,94	0,51	1,00	> 0,10	> 0,10	> 0,10
**hsa-miR-92a-2-5p**	34,10	34,11	35,39	33,93	1,52	1,02	0,55	1,00	> 0,10	> 0,10	> 0,10
**hsa-miR-92a-3p**	25,70	25,03	25,48	24,57	0,78	0,84	0,81	1,00	> 0,10	> 0,10	> 0,10
**hsa-miR-92b-3p**	36,37	35,42	34,50	35,39	0,60	1,02	2,53	1,00	> 0,10	> 0,10	> 0,10
**hsa-miR-93-3p**	31,78	30,94	31,66	30,71	0,82	0,99	0,79	1,00	> 0,10	> 0,10	> 0,10
**hsa-miR-934**	34,12	34,96	33,69	34,51	1,54	0,77	1,74	1,00	> 0,10	> 0,10	> 0,10
**hsa-miR-93-5p**	26,05	25,35	26,09	25,26	0,99	1,09	0,85	1,00	> 0,10	> 0,10	> 0,10
**hsa-miR-937-3p**	35,67	35,85	36,40	35,49	2,58	0,90	0,68	1,00	> 0,10	> 0,10	> 0,10
**hsa-miR-9-3p**	**40,00**	36,13	36,42	**40,00**	1,71	16,95*	13,05	1,00	> 0,10	**0,0267**	> 0,10
**hsa-miR-940**	28,72	28,75	29,70	28,84	2,14	1,24	0,84	1,00	0,0575	> 0,10	> 0,10
**hsa-miR-941**	32,03	31,50	33,10	30,90	0,78	0,77	0,33	1,00	> 0,10	> 0,10	> 0,10
**hsa-miR-942-5p**	35,94	36,97	36,71	35,48	1,05	0,39	0,60	1,00	> 0,10	> 0,10	> 0,10
**hsa-miR-95-3p**	33,46	31,54	32,51	31,56	0,46	1,18	0,78	1,00	> 0,10	> 0,10	> 0,10
**hsa-miR-9-5p**	36,13	33,51	34,43	34,61	0,46	2,53	1,75	1,00	> 0,10	> 0,10	> 0,10
**hsa-miR-96-5p**	28,93	28,04	28,63	28,64	1,40	1,77	1,53*	1,00	> 0,10	0,0570	**0,0430**
**hsa-miR-98-5p**	31,14	30,17	30,62	30,25	0,93	1,23	1,18	1,00	> 0,10	> 0,10	> 0,10

**hsa-miR-99a-3p**	30,40	29,29	30,34	29,26	0,78	1,14	0,72	1,00	> 0,10	> 0,10	0,0738
**hsa-miR-99a-5p**	23,74	22,59	23,65	22,34	0,65	0,98	0,61*	1,00	> 0,10	> 0,10	**0,0109**
**hsa-miR-99b-3p**	33,33	32,38	32,49	32,15	0,76	0,99	1,20	1,00	> 0,10	> 0,10	> 0,10
**hsa-miR-99b-5p**	27,71	26,79	27,27	26,68	0,84	1,08	1,01	1,00	> 0,10	> 0,10	> 0,10

Supplementary Table S4. Identification of target genes and pathways potentially altered by the miRNA signature performed using the miRNet web-based platform

No.	miRNet Pathway	Hits	P-value
1	Pathways in cancer	53	7.72e-8
2	Chronic myeloid leukemia	21	0.00000132
3	HTLV-I infection	34	0.0000389
4	Pancreatic cancer	18	0.0000389
5	Prostate cancer	20	0.0000652
6	Neurotrophin signaling pathway	24	0.000114
7	Cell cycle	23	0.000382
8	Glioma	15	0.000816
9	MAPK signaling pathway	36	0.00175
10	Colorectal cancer	12	0.00208
11	Small cell lung cancer	16	0.00208
12	Focal adhesion	28	0.0052
13	Chagas disease (American trypanosomiasis)	16	0.00643
14	Epstein-Barr virus infection	16	0.00775
15	TGF-beta signaling pathway	15	0.00869
16	Measles	17	0.00869
17	p53 signaling pathway	13	0.00885
18	Renal cell carcinoma	12	0.00885
19	Non-small cell lung cancer	11	0.00885
20	Tight junction	18	0.0142
21	Vibrio cholerae infection	6	0.0147
22	Melanogenesis	16	0.0159

23	Toxoplasmosis	15	0.0175
24	ErbB signaling pathway	14	0.0232
25	Thyroid cancer	7	0.0232
26	Chemokine signaling pathway	24	0.024
27	Adherens junction	12	0.024
28	Endometrial cancer	9	0.024
29	Bladder cancer	7	0.0241
30	Wnt signaling pathway	19	0.0384
31	Dopaminergic synapse	17	0.0389
32	Insulin signaling pathway	18	0.0467
33	Toll-like receptor signaling pathway	14	0.048
34	Osteoclast differentiation	16	0.0543
35	Endocrine and other factor-regulated calcium reabsorption	7	0.0698
36	Amphetamine addiction	10	0.0776
37	GnRH signaling pathway	13	0.0793
38	B cell receptor signaling pathway	11	0.0842
39	Basal cell carcinoma	8	0.0855
40	Acute myeloid leukemia	9	0.091
41	Fc gamma R-mediated	13	0.0916
42	Vascular smooth muscle contraction	14	0.0963
43	NOD-like receptor signaling pathway	8	0.0963
44	Melanoma	10	0.0963
45	Jak-STAT signaling pathway	13	0.0976
46	Pertussis	8	0.128

47	Apoptosis	11	0.136
48	Viral myocarditis	5	0.145
49	Cholinergic synapse	12	0.146
50	Axon guidance	14	0.15
51	Fc epsilon RI signaling pathway	10	0.15
52	Influenza A	13	0.15
53	Leukocyte transendothelial	13	0.157
54	Arrhythmogenic right ventricular cardiomyopathy (ARVC)	3	0.189
55	Type II diabetes mellitus	7	0.191
56	Regulation of actin cytoskeleton	19	0.2
57	Long-term potentiation	9	0.201
58	Long-term depression	9	0.201
59	Pancreatic secretion	5	0.201
60	Prion diseases	4	0.201
61	Leishmaniasis	7	0.227
62	Cocaine addiction	6	0.273
63	T cell receptor signaling pathway	11	0.279
64	VEGF signaling pathway	9	0.283
65	mTOR signaling pathway	6	0.291
66	Hedgehog signaling pathway	7	0.291
67	Gap junction	10	0.291
68	Bacterial invasion of epithelial cells	7	0.291
69	Hepatitis C	11	0.291
70	Hypertrophic cardiomyopathy	4	0.291

71	Dilated cardiomyopathy	9	0.291
72	Sphingolipid metabolism	6	0.299
73	Amoebiasis	6	0.299
74	Progesterone-mediated oocyte maturation	9	0.313
75	Inositol phosphate metabolism	7	0.314
76	Shigellosis	6	0.314
77	SNARE interactions in vesicular transport	4	0.329
78	Protein processing in endoplasmic reticulum	13	0.329
79	Synaptic vesicle cycle	3	0.344
80	Salivary secretion	6	0.344
81	Carbohydrate digestion and absorption	3	0.344
82	Oocyte meiosis	11	0.358
83	Bile secretion	4	0.373
84	Legionellosis	5	0.378
85	Adipocytokine signaling pathway	7	0.386
86	Pentose and glucuronate interconversions	4	0.394
87	Circadian rhythm - mammal	3	0.483
88	Vasopressin-regulated water reabsorption	3	0.483
89	Gastric acid secretion	6	0.483
90	Selenocompound metabolism	2	0.488
91	Dorso-ventral axis formation	2	0.488

92	Cysteine and methionine metabolism	4	0.504
93	Pathogenic Escherichia coli infection	4	0.535
94	Retrograde endocannabinoid signaling	6	0.563
95	African trypanosomiasis	3	0.583
96	Epithelial cell signaling in Helicobacter pylori infection	4	0.589
97	Nicotinate and nicotinamide metabolism	3	0.6
98	Phosphatidylinositol signaling system	7	0.6
99	Serotonergic synapse	8	0.6
100	Alcoholism	14	0.6
101	Herpes simplex infection	9	0.64
102	Phenylalanine, tyrosine and tryptophan biosynthesis	1	0.65
103	Huntington's disease	3	0.662
104	Primary bile acid biosynthesis	2	0.706
105	Morphine addiction	5	0.711
106	Fatty acid biosynthesis	1	0.719
107	Glycerophospholipid metabolism	7	0.719
108	Natural killer cell mediated cytotoxicity	11	0.758
109	Transcriptional misregulation in cancer	2	0.77
110	Rheumatoid arthritis	2	0.77
111	Salmonella infection	6	0.772

112	Aminoacyl-tRNA biosynthesis	1	0.775
113	Pyrimidine metabolism	8	0.78
114	Cytokine-cytokine receptor interaction	19	0.78
115	Cytosolic DNA-sensing pathway	2	0.78
116	Glutamatergic synapse	8	0.78
117	alpha-Linolenic acid metabolism	2	0.799
118	Regulation of autophagy	1	0.799
119	Lysosome	1	0.799
120	Mineral absorption	1	0.799
121	Taurine and hypotaurine metabolism	1	0.863
122	Fatty acid elongation	2	0.868
123	Glycerolipid metabolism	4	0.886
124	Complement and coagulation cascades	5	0.89
125	Maturity onset diabetes of the young	2	0.89
126	Fatty acid metabolism	3	0.907
127	Butanoate metabolism	2	0.907
128	Ribosome biogenesis in eukaryotes	4	0.907
129	RNA transport	9	0.907
130	ECM-receptor interaction	6	0.907
131	Staphylococcus aureus infection	3	0.907
132	Pyruvate metabolism	3	0.918
133	Pentose phosphate pathway	2	0.939

134	Cardiac muscle contraction	1	0.945
135	Citrate cycle (TCA cycle)	2	0.997
136	Mucin type O-Glycan biosynthesis	2	0.997
137	Cell adhesion molecules (CAMs)	8	0.997
138	Taste transduction	3	0.997
139	Glycolysis / Gluconeogenesis	1	1
140	Fructose and mannose metabolism	2	1
141	Galactose metabolism	1	1
142	Steroid biosynthesis	1	1
143	Steroid hormone biosynthesis	2	1
144	Purine metabolism	8	1
145	Alanine, aspartate and glutamate metabolism	2	1
146	Glycine, serine and threonine metabolism	1	1
147	Lysine degradation	3	1
148	Arginine and proline metabolism	2	1
149	Tyrosine metabolism	1	1
150	Phenylalanine metabolism	1	1
151	Tryptophan metabolism	1	1
152	Starch and sucrose metabolism	2	1
153	N-Glycan biosynthesis	2	1
154	Amino sugar and nucleotide sugar metabolism	2	1
155	Glycosaminoglycan degradation	1	1

156	Glycosylphosphatidylinositol(GPI)-anchor biosynthesis	1	1
157	Ether lipid metabolism	2	1
158	Arachidonic acid metabolism	3	1
159	Linoleic acid metabolism	1	1
160	Propanoate metabolism	1	1
161	One carbon pool by folate	1	1
162	Pantothenate and CoA biosynthesis	1	1
163	Retinol metabolism	2	1
164	Porphyrin and chlorophyll metabolism	2	1
165	Terpenoid backbone biosynthesis	1	1
166	Metabolism of xenobiotics by cytochrome P450	1	1
167	Drug metabolism - cytochrome P450	1	1
168	Drug metabolism - other enzymes	2	1
169	mRNA surveillance pathway	4	1
170	RNA degradation	3	1
171	PPAR signaling pathway	4	1
172	Fanconi anemia pathway	1	1
173	Calcium signaling pathway	11	1
174	Endocytosis	5	1
175	Phagosome	2	1
176	Notch signaling pathway	2	1
177	Antigen processing and presentation	1	1

178	RIG-I-like receptor signaling pathway	3	1
179	GABAergic synapse	4	1
180	Olfactory transduction	3	1
181	Phototransduction	1	1
182	Aldosterone-regulated sodium reabsorption	2	1
183	Alzheimer's disease	3	1
184	Amyotrophic lateral sclerosis (ALS)	2	1
185	Tuberculosis	9	1
186	Systemic lupus erythematosus	1	1

Supplementary Table S5. Nanoparticle tracking analysis of semen vesicles using a Nanosight device.

A.

	HCt	BPH	PCa-noV	PCa-V
**Size mean (nm)**	128,00 ± 5,20	135,47 ± 9,18	137,73 ± 8,11	142,47 ± 8,16
**Size mode (nm)**	100,57 ± 2,94	105,10 ± 1,73	108,57 ± 3,92	115,97 ± 2,72
**Size D10 (nm)**	77,23 ± 3,27	82,60 ± 3,75	83,63 ± 3,97	81,97 ± 5,67
**Size D50 (nm)**	105,57 ± 3,65	112,97 ± 6,06	116,07 ± 4,97	116,63 ± 5,35
**Size D90 (nm)**	169,40 ± 13,31	177,37 ± 19,68	183,23 ± 16,51	205,97 ± 34,02

B.

	Percentile undersize			
**Bin centre (nm)**	**HCt (%)**	**BPH (%)**	**PCa-noV (%)**	**PCa-V (%)**
5	0 ± 0	0 ± 0	0 ± 0	0 ± 0
15	0 ± 0	1,62E-10 ± 2,81E-10	2,26E-11 ± 3,92E-11	1,641E-09 ± 2,84E-09
25	3,67E-10 ± 6,36E-10	1,24E-03 ± 2,15E-03	1,01E-07 ± 1,74E-07	8,84E-07 ± 1,53E-06
35	1E-3 ± 2E-3	1,97E-03 ± 3,41E-03	2,20E-06 ± 1,92E-06	6,07E-05 ± 1,05E-04
45	2E-3 ± 3E-3	2,04E-03 ± 3,43E-03	1,41E-03 ± 1,40E-03	0,08 ± 0,11
55	0,01 ± 0,01	8,09E-03 ± 6,57E-03	0,02 ± 0,01	0,10 ± 0,09
65	0,43 ± 0,36	0,17 ± 0,09	0,16 ± 0,10	0,22 ± 0,16
75	3,55 ± 1,26	1,36 ± 0,96	1,35 ± 0,80	1,80 ± 1,48
85	13,71 ± 4,69	7,21 ± 3,69	6,40 ± 3,37	8,20 ± 6,11
95	27,80 ± 4,94	20,37 ± 5,41	18,28 ± 6,11	20,23 ± 6,88
105	43,53 ± 3,32	35,95 ± 4,71	30,72 ± 5,62	31,25 ± 6,22
115	55,79 ± 4,39	48,29 ± 5,22	42,90 ± 4,97	41,77 ± 5,54
125	64,58 ± 5,50	57,44 ± 7,14	54,40 ± 5,39	52,80 ± 5,20
135	72,02 ± 5,13	65,24 ± 8,57	64,32 ± 6,02	62,35 ± 5,89
145	79,11 ± 4,18	72,68 ± 8,56	71,73 ± 5,80	69,15 ± 6,46
155	84,72 ± 3,18	79,36 ± 7,37	77,17 ± 4,65	73,81 ± 6,94
165	88,44 ± 2,39	84,33 ± 6,02	81,75 ± 3,70	78,33 ± 7,40
175	90,88 ± 2,37	87,77 ± 4,95	85,90 ± 3,32	82,61 ± 6,67
185	92,59 ± 2,59	90,31 ± 4,12	89,52 ± 3,45	85,99 ± 5,03
195	93,82 ± 2,58	92,26 ± 3,37	92,19 ± 3,72	88,44 ± 4,19
205	94,72 ± 2,28	93,69 ± 2,72	93,81 ± 3,71	90,39 ± 4,36
215	95,45 ± 1,75	94,72 ± 2,23	94,79 ± 3,42	91,75 ± 4,56
225	96,03 ± 1,14	95,48 ± 1,88	95,52 ± 3,01	92,61 ± 4,48
235	96,48 ± 0,76	96,01 ± 1,68	96,16 ± 2,59	93,35 ± 4,37
245	96,86 ± 0,68	96,41 ± 1,56	96,74 ± 2,22	94,10 ± 4,15
255	97,28 ± 0,72	96,76 ± 1,46	97,25 ± 1,90	94,89 ± 3,61
265	97,72 ± 0,82	97,11 ± 1,38	97,72 ± 1,57	95,75 ± 2,79
275	98,07 ± 0,89	97,45 ± 1,32	98,22 ± 1,25	96,57 ± 1,90
285	98,28 ± 0,89	97,78 ± 1,29	98,68 ± 0,96	97,21 ± 1,17
295	98,42 ± 0,86	98,11 ± 1,24	98,96 ± 0,71	97,63 ± 0,67
305	98,53 ± 0,82	98,46 ± 1,12	99,11 ± 0,55	97,89 ± 0,36
315	98,65 ± 0,78	98,80 ± 0,91	99,22 ± 0,49	98,06 ± 0,19
325	98,79 ± 0,74	99,05 ± 0,73	99,32 ± 0,49	98,20 ± 0,11
335	98,93 ± 0,68	99,19 ± 0,61	99,41 ± 0,52	98,36 ± 0,08
345	99,10 ± 0,56	99,28 ± 0,55	99,48 ± 0,54	98,54 ± 0,15
355	99,29 ± 0,37	99,36 ± 0,49	99,54 ± 0,51	98,73 ± 0,22
365	99,47 ± 0,19	99,43 ± 0,44	99,60 ± 0,42	98,92 ± 0,29
375	99,58 ± 0,10	99,49 ± 0,39	99,66 ± 0,33	99,14 ± 0,35
385	99,67 ± 0,07	99,55 ± 0,34	99,70 ± 0,27	99,33 ± 0,26
395	99,73 ± 0,11	99,59 ± 0,29	99,72 ± 0,24	99,48 ± 0,15
405	99,77 ± 0,14	99,64 ± 0,25	99,74 ± 0,21	99,58 ± 0,10
415	99,80 ± 0,15	99,68 ± 0,21	99,75 ± 0,19	99,67 ± 0,08
425	99,83 ± 0,14	99,72 ± 0,19	99,77 ± 0,17	99,75 ± 0,11
435	99,87 ± 0,10	99,75 ± 0,18	99,77 ± 0,16	99,81 ± 0,13

445	99,92 ± 0,05	99,79 ± 0,16	99,78 ± 0,15	99,84 ± 0,13
455	99,96 ± 0,02	99,82 ± 0,13	99,78 ± 0,14	99,86 ± 0,12
465	99,98 ± 0,02	99,85 ± 0,09	99,79 ± 0,14	99,87 ± 0,12
475	99,98 ± 0,02	99,88 ± 0,06	99,79 ± 0,13	99,88 ± 0,11
485	99,98 ± 0,02	99,90 ± 0,05	99,79 ± 0,13	99,89 ± 0,11
495	99,98 ± 0,02	99,92 ± 0,05	99,79 ± 0,13	99,90 ± 0,11
505	99,98 ± 0,02	99,93 ± 0,06	99,80 ± 0,12	99,91 ± 0,12
515	99,98 ± 0,02	99,94 ± 0,06	99,80 ± 0,12	99,91 ± 0,12
525	99,98 ± 0,02	99,94 ± 0,06	99,80 ± 0,11	99,92 ± 0,12
535	99,98 ± 0,02	99,95 ± 0,06	99,81 ± 0,09	99,92 ± 0,13
545	99,98 ± 0,02	99,95 ± 0,06	99,83 ± 0,07	99,92 ± 0,13
555	99,98 ± 0,02	99,95 ± 0,06	99,85 ± 0,05	99,92 ± 0,13
565	99,98 ± 0,02	99,96 ± 0,06	99,87 ± 0,05	99,92 ± 0,13
575	99,98 ± 0,02	99,96 ± 0,05	99,90 ± 0,06	99,92 ± 0,13
585	99,98 ± 0,02	99,97 ± 0,04	99,92 ± 0,07	99,92 ± 0,13
595	99,98 ± 0,02	99,98 ± 0,03	99,94 ± 0,07	99,92 ± 0,13
605	99,98 ± 0,02	99,99 ± 0,01	99,95 ± 0,07	99,92 ± 0,13
615	99,98 ± 0,02	100 ± 0	99,95 ± 0,07	99,92 ± 0,13
625	99,98 ± 0,02	100 ± 0	99,96 ± 0,06	99,92 ± 0,13
635	99,98 ± 0,01	100 ± 0	99,97 ± 0,06	99,92 ± 0,13
645	99,99 ± 0,01	100 ± 0	99,97 ± 0,05	99,93 ± 0,13
655	99,99 ± 0,01	100 ± 0	99,98 ± 0,04	99,93 ± 0,13
665	99,99 ± 0,01	100 ± 0	99,98 ± 0,03	99,93 ± 0,13
675	99,99 ± 0,01	100 ± 0	99,99 ± 0,02	99,93 ± 0,12
685	99,99 ± 0,01	100 ± 0	99,99 ± 0,02	99,93 ± 0,12
695	99,99 ± 0,01	100 ± 0	99,99 ± 0,01	99,94 ± 0,11
705	99,99 ± 0,01	100 ± 0	100 ± 0	99,96 ± 0,09
715	100 ± 0	100 ± 0	100 ± 0	99,96 ± 0,08
725	100 ± 0	100 ± 0	100 ± 0	99,97 ± 0,05
735	100 ± 0	100 ± 0	100 ± 0	99,98 ± 0,04
745	100 ± 0	100 ± 0	100 ± 0	99,99 ± 0,02
755	100 ± 0	100 ± 0	100 ± 0	99,99 ± 0,01
765	100 ± 0	100 ± 0	100 ± 0	100 ± 0
775	100 ± 0	100 ± 0	100 ± 0	100 ± 0
785	100 ± 0	100 ± 0	100 ± 0	100 ± 0
795	100 ± 0	100 ± 0	100 ± 0	100 ± 0
805	100 ± 0	100 ± 0	100 ± 0	100 ± 0
815	100 ± 0	100 ± 0	100 ± 0	100 ± 0
825	100 ± 0	100 ± 0	100 ± 0	100 ± 0
835	100 ± 0	100 ± 0	100 ± 0	100 ± 0
845	100 ± 0	100 ± 0	100 ± 0	100 ± 0
855	100 ± 0	100 ± 0	100 ± 0	100 ± 0
865	100 ± 0	100 ± 0	100 ± 0	100 ± 0
875	100 ± 0	100 ± 0	100 ± 0	100 ± 0
885	100 ± 0	100 ± 0	100 ± 0	100 ± 0
895	100 ± 0	100 ± 0	100 ± 0	100 ± 0
905	100 ± 0	100 ± 0	100 ± 0	100 ± 0
915	100 ± 0	100 ± 0	100 ± 0	100 ± 0
925	100 ± 0	100 ± 0	100 ± 0	100 ± 0
935	100 ± 0	100 ± 0	100 ± 0	100 ± 0
945	100 ± 0	100 ± 0	100 ± 0	100 ± 0
955	100 ± 0	100 ± 0	100 ± 0	100 ± 0
965	100 ± 0	100 ± 0	100 ± 0	100 ± 0
975	100 ± 0	100 ± 0	100 ± 0	100 ± 0
985	100 ± 0	100 ± 0	100 ± 0	100 ± 0
995	100 ± 0	100 ± 0	100 ± 0	100 ± 0

Supplementary Table S6. List of the 50 miRNAs showing the most stable expression among samples

miRNAs	PCa-V1 *Cp* value	PCa-V2 *Cp* value	PCa-V3 *Cp* value	PCa-noV1 *Cp* value	PCa-noV2 *Cp* value	PCa-noV3 *Cp* value	BPH-noV1 *Cp* value	BPH-noV2 *Cp* value	BPH-noV3 *Cp* value	HCt-noV1 *Cp value*	HCt-noV2 *Cp value*	HCt-noV3 *Cp value*	mean (*Cp* value)	SD (*Cp* value)	CV (*Cp* value)
average 50 stables miRNAs (selected to normalize)	31,57	30,43	30,41	29,81	30,67	30,24	31,12	30,60	30,15	29,73	30,27	30,07	30,42	0,52	0,0172
hsa-miR-126-3p	30,89	29,83	29,50	29,29	30,30	29,30	29,88	29,92	29,67	29,63	29,09	29,72	29,75	0,49	0,0163
hsa-miR-30e-3p	29,00	28,11	28,06	27,32	28,35	27,91	28,79	28,14	28,03	27,51	27,88	27,58	28,05	0,49	0,0174
**hsa-miR-769-5p**	32,50	32,23	31,25	31,18	32,16	31,02	32,67	31,85	31,57	31,25	31,56	31,33	31,71	0,56	0,0175
**hsa-miR-423-5p**	28,21	27,53	27,78	26,48	27,68	27,62	28,13	27,85	27,54	26,91	27,55	27,18	27,54	0,49	0,0178
**hsa-miR-491-5p**	32,30	31,36	30,27	30,56	31,58	30,94	31,26	31,60	30,76	30,73	30,80	30,81	31,08	0,56	0,0180
**hsa-miR-152-3p**	29,54	28,10	28,65	27,94	28,72	28,47	28,76	28,62	27,80	27,96	27,71	28,02	28,36	0,53	0,0188
**hsa-miR-2110**	32,21	32,12	31,09	30,57	32,05	31,64	32,81	32,16	31,82	31,21	31,77	31,28	31,73	0,61	0,0192
**hsa-miR-196b-3p**	32,77	31,29	31,20	31,46	31,63	31,47	32,70	32,77	31,88	31,25	31,29	31,78	31,79	0,61	0,0193
**hsa-miR-219a-5p**	35,00	33,44	33,04	33,52	33,48	34,02	34,79	33,76	33,48	33,05	34,03	32,95	33,71	0,65	0,0194
**hsa-miR-21-3p**	34,46	33,78	33,11	33,17	34,57	34,09	34,10	33,98	32,83	32,57	33,13	33,91	33,64	0,66	0,0195
**hsa-miR-148b-3p**	28,97	27,50	27,59	27,09	27,84	27,29	28,27	27,81	27,23	26,99	27,49	27,49	27,63	0,55	0,0198
**hsa-miR-140-5p**	32,32	31,04	30,61	30,78	31,18	30,49	31,50	31,64	30,56	30,01	30,95	30,72	30,98	0,61	0,0198
**hsa-miR-744-5p**	31,74	31,59	31,06	30,25	31,89	30,98	31,67	31,89	30,84	30,01	31,03	30,81	31,15	0,62	0,0201
**hsa-miR-185-5p**	28,69	27,62	27,54	26,66	27,97	27,27	27,87	27,60	27,23	26,93	27,08	26,93	27,45	0,56	0,0203
**hsa-miR-200a-5p**	32,76	31,22	31,22	30,77	31,66	30,89	31,76	31,01	30,87	30,23	30,90	30,99	31,19	0,64	0,0204
**hsa-miR-26b-3p**	34,34	33,90	33,23	32,94	33,19	32,85	34,47	34,47	33,01	32,58	33,17	32,88	33,42	0,68	0,0204
**hsa-miR-22-5p**	30,76	29,57	29,71	28,99	30,33	29,53	30,08	29,72	28,72	28,80	29,52	30,02	29,64	0,61	0,0205
**hsa-miR-30d-3p**	32,49	31,47	31,75	31,03	32,22	31,10	32,30	31,99	31,86	30,32	31,53	30,88	31,58	0,65	0,0205
**hsa-let-7d-5p**	29,02	28,09	28,06	26,84	28,13	27,98	28,21	27,89	27,52	27,06	27,68	27,47	27,83	0,57	0,0206
**hsa-miR-99b-5p**	28,33	27,30	27,50	26,47	27,21	26,67	27,74	27,30	26,75	26,58	26,94	26,53	27,11	0,56	0,0208
**hsa-miR-18b-5p**	30,98	29,83	29,43	28,96	30,10	29,25	30,33	29,98	29,44	28,90	29,32	29,19	29,64	0,62	0,0208
**hsa-miR-149-3p**	31,99	32,24	32,99	31,70	33,03	32,49	33,92	32,57	33,02	31,57	32,51	31,76	32,48	0,69	0,0211
**hsa-miR-182-5p**	28,82	27,61	27,69	26,54	27,69	27,16	28,21	27,68	27,50	27,47	27,48	26,87	27,56	0,58	0,0212
**hsa-miR-101-5p**	34,53	33,17	32,98	32,80	33,85	32,67	34,34	33,26	33,12	32,55	33,54	32,21	33,25	0,71	0,0212
**hsa-miR-339-3p**	32,07	31,00	30,19	30,23	31,23	30,19	31,69	31,21	30,56	30,22	30,77	30,12	30,79	0,65	0,0213
**hsa-miR-98-5p**	31,78	30,73	30,91	29,46	30,88	30,17	31,24	30,31	30,31	29,84	30,92	30,00	30,55	0,65	0,0213
**hsa-miR-140-3p**	29,86	28,61	28,37	27,86	28,81	28,64	29,07	28,80	28,17	27,58	28,13	28,10	28,50	0,61	0,0213
**hsa-miR-425-3p**	32,60	31,27	30,88	30,60	31,63	31,37	31,65	30,84	30,83	30,08	30,64	30,67	31,09	0,66	0,0213
**hsa-miR-374b-3p**	35,45	33,54	33,96	34,25	33,79	33,90	34,11	34,99	34,01	32,52	33,53	33,74	33,98	0,73	0,0215
**hsa-miR-194-5p**	31,19	29,76	30,11	28,91	29,68	29,08	29,85	29,50	29,77	28,69	29,48	29,61	29,64	0,64	0,0215
**hsa-miR-106b-3p**	34,00	32,67	32,12	31,77	32,63	32,73	33,63	32,72	32,22	31,75	32,57	31,82	32,55	0,70	0,0216
**hsa-miR-590-5p**	30,73	28,95	29,19	28,76	29,14	28,80	29,77	29,64	28,94	28,62	28,67	28,55	29,15	0,63	0,0216

**hsa-miR-183-5p**	30,49	29,10	29,26	27,97	28,90	28,75	29,18	28,85	28,50	28,74	29,17	28,30	28,93	0,62	0,0216
**hsa-miR-7-5p**	33,47	31,76	33,13	31,54	31,34	32,26	33,10	31,92	32,11	31,55	32,63	32,06	32,24	0,70	0,0216
**hsa-miR-128-3p**	32,80	30,86	31,10	30,11	30,77	30,75	31,46	31,00	30,79	30,54	31,49	31,44	31,09	0,67	0,0216
**hsa-miR-328-3p**	32,60	30,94	30,99	29,91	31,58	31,18	31,61	31,72	30,81	30,93	30,88	30,58	31,15	0,67	0,0217
**hsa-miR-20a-3p**	31,50	30,85	30,66	30,22	30,75	29,84	31,89	30,47	30,78	29,54	30,47	29,99	30,58	0,66	0,0217
**hsa-miR-320c**	30,06	29,32	29,72	28,26	29,50	29,59	30,38	29,37	28,95	28,27	29,07	28,98	29,29	0,64	0,0218
**hsa-miR-20b-3p**	32,64	31,26	31,66	31,03	31,64	30,96	32,47	31,85	30,87	30,20	31,09	31,25	31,41	0,69	0,0219
**hsa-miR-151a-3p**	29,75	28,59	28,89	28,10	28,88	28,26	29,24	28,71	28,07	27,59	28,00	27,90	28,50	0,63	0,0219
**hsa-let-7d-3p**	31,09	29,30	29,69	29,32	29,94	29,44	30,49	30,49	29,05	29,23	29,85	29,02	29,74	0,66	0,0220
**hsa-miR-320a**	27,08	26,02	26,45	25,12	26,11	26,20	26,72	26,08	25,71	25,16	25,65	25,95	26,02	0,58	0,0221
**hsa-miR-130b-5p**	35,54	33,86	33,77	33,98	34,63	35,64	35,78	33,91	34,02	34,13	35,24	34,34	34,57	0,77	0,0222
**hsa-let-7i-5p**	29,64	28,39	28,60	27,33	28,62	28,31	28,69	28,20	27,49	27,59	28,07	28,14	28,25	0,63	0,0222
**hsa-miR-210-3p**	30,79	29,50	29,44	28,18	29,59	28,95	29,66	29,69	29,06	28,82	29,11	28,75	29,29	0,65	0,0222
**hsa-miR-628-3p**	34,00	33,41	34,47	33,36	33,15	33,52	35,31	32,61	33,00	32,80	33,82	33,52	33,58	0,75	0,0223
**hsa-miR-18a-5p**	32,02	30,59	29,99	29,74	30,35	29,59	30,96	30,57	30,16	29,64	29,98	30,11	30,31	0,68	0,0224
**hsa-let-7b-3p**	32,71	31,21	31,30	30,69	31,58	30,80	32,45	31,63	30,85	30,64	31,03	30,52	31,28	0,70	0,0225
**hsa-miR-15b-3p**	35,10	33,95	34,24	35,44	35,66	33,78	34,22	33,49	33,53	34,61	34,05	35,46	34,46	0,78	0,0225
**hsa-miR-191-5p**	27,01	26,01	26,15	25,12	26,04	26,09	26,90	26,21	26,01	25,24	25,50	25,45	25,98	0,59	0,0226

Data from the 50 most stable miRNAs were selected to normalize data from miRNA profiling

hsa-miR-30e-3p and hsa-miR-126-3p (in italic) were selected to normalize data from candidate miRNAs

PCa-V: prostate cancer in vasectomized man; PCa-noV : prostate cancer in non-vasectomized man; BPH-noV: benign prostate hyperplasia in non-vasectomized man;

HCt-noV: healthy non-vasectomized man as control

Supplementary Table S7. List of the miRNA PCR primers and conditions for real-time PCR.

miRNA	5'-3' sequence	ID[a]	qPCR cycle conditions	
hsa-miR-126-3p	UCGUACCGUGAGUAAUAAUGCG	YP00204227	Polymerase Activation/ Denaturation	95°C, 10 min
hsa-miR-128-3p	UCACAGUGAACCGGUCUCUUU	YP00205995		
hsa-miR-130a-3p	CAGUGCAAUGUUAAAAGGGCAU	YP00204658	2 Step Amplification	45 amplification cycles: 95°C, 10 s 60°C, 1 min (Ramp-rate 1.6°C/s)
hsa-miR-142-3p	UGUAGUGUUUCCUACUUUAUGGA	YP00204291		
hsa-miR-142-5p	CAUAAAGUAGAAAGCACUACU	YP00204722		
hsa-miR-150-5p	UCUCCCAACCCUUGUACCAGUG	YP00204660		
hsa-miR-182-3p	UGGUUCUAGACUUGCCAACUA	YP00204098		
hsa-miR-187-5p	GGCUACAACACAGGACCCGGGC	YP00205920		
hsa-miR-212-5p	ACCUUGGCUCUAGACUGCUUACU	YP00205401		
hsa-miR-217	UACUGCAUCAGGAACUGAUUGGA	YP00204010		
hsa-miR-222-3p	AGCUACAUCUGGCUACUGGGU	YP00204551		
hsa-miR-223-3p	UGUCAGUUUGUCAAAUACCCCA	YP00205986		
hsa-miR-30e-3p	CUUUCAGUCGGAUGUUUACAGC	YP00204410		
hsa-miR-342-3p	UCUCACACAGAAAUCGCACCCGU	YP00205625		
hsa-miR-370-3p	GCCUGCUGGGGUGGAACCUGGU	YP00204011		
hsa-miR-374b-5p	AUAUAAUACAACCUGCUAAGUG	YP00204608		

[a]*miRCURY LNA*^{TM} *miRNA PCR Assay* (Qiagen)
